# Supplementary material for: Hyperthermophilic methanogenic archaea act as high-pressure CH4 cell factories
Source: Commun Biol. 2021 Mar 5;4:289. doi: 10.1038/s42003-021-01828-5 (PMC7935968; doi:10.1038/s42003-021-01828-5)
Supplement: Supplementary file 2 — Supplementary Information [file 42003_2021_1828_MOESM2_ESM.pdf]

Supplementary Materials

Hyperthermophilic methanogenic archaea act as high-pressure CH_4 cell factories

Lisa-Maria Mauerhofer[1], Sara Zwirtmayr[2], Patricia Pappenreiter[2], Sébastien Bernacchi[3], Arne H. Seifert[3], Barbara Reischl[1,3], Tilman Schmider[1], Ruth-Sophie Taubner[1,2], Christian Paulik[2], Simon K.-M. R. Rittmann[1,*]

[1]Archaea Physiology & Biotechnology Group, Department Functional and Evolutionary Ecology, Universität Wien, Wien, Austria
[2]Institute for Chemical Technology of Organic Materials, Johannes Kepler University Linz, Linz, Austria
[3]Krajete GmbH, Linz, Austria

[*]Corresponding author:
Dr. Simon K.-M. R. Rittmann, Privatdoz.
Archaea Physiology & Biotechnology Group
Department of Functional and Evolutionary Ecology
Universität Wien
Althanstraße 14
1090 Wien
Austria
Tel.: +43-4277-76513
eFax.: +43-4277-876513
Email: simon.rittmann@univie.ac.at

Media compositions

141 medium KCl 0.34 g L^{-1}, $MgCl_2 \cdot 6H_2O$ 4 g L^{-1}, $MgSO_4 \cdot 7H_2O$ 3.45 g L^{-1}, NH_4Cl 0.25 g L^{-1}, $CaCl_2 \cdot 2H_2O$ 0.14 g L^{-1}, K_2HPO_4 0.14 g L^{-1}, NaCl 18 g L^{-1}, TE solution (141 medium) 10 mL L^{-1}, $FeII(NH_4)_2(SO_4)_2 \cdot 6H_2O$ solution (w:v = 0.1%) 2 mL L^{-1}, Na-acetate 1 mL L^{-1}, Yeast extract 2 g L^{-1}, $NaHCO_3$ 5 g L^{-1}, vitamin solution 10 mL L^{-1}, L-Cysteine-HCl·H_2O 0.5 g L^{-1}, $Na_2S \cdot 9H_2O$ 0.5 g L^{-1}. Trace element (TE) solution for 141-medium contained the following (TES1): nitrilotriacetic acid 1.5 g L^{-1}, $MgSO_4 \cdot 7H_2O$ 3 g L^{-1}, $MnCl_2 \cdot 4H_2O$ 0.585 g L^{-1}, NaCl 1 g L^{-1}, $FeSO_4 \cdot 7H_2O$ 0.1 g L^{-1}, $CoSO_4 \cdot 7H_2O$ 0.18 g L^{-1}, $CaCl_2 \cdot 2H_2O$ 0.1 g L^{-1}, $ZnSO_4 \cdot 7H_2O$ 0.18 g L^{-1}, $CuSO_4$ 0.006 g L^{-1}, $KAl(SO_4)_2 \cdot 12H_2O$ 0.02 g L^{-1}, H_3BO_3 0.01 g L^{-1}, $Na_2MoO_4 \cdot 2H_2O$ 0.01g L^{-1}, $NiCl_2 \cdot 6H_2O$ 0.03 g L^{-1}, $Na_2SeO_3 \cdot 5H_2O$ 0.3 mg L^{-1}, $Na_2WO_4 \cdot 2H_2O$ 0.4 mg L^{-1}. Wolf´s vitamin solution (vitamin solution of 141 medium) contained the following: biotine 20 mg L^{-1} (81.9 μM), folic acid 20 mg L^{-1} (45.3 μM), pyridoxamine dihydrochloride 100 mg L^{-1} (386.0 μM), thiamine hydrochloride 50 mg L^{-1} (148.0 μM), riboflavin 50 mg L^{-1} (133.0 μM), nicotinic acid 50 mg L^{-1} (406.0 μM), DL-calcium pantothenate 50 mg L^{-1} (105.0 μM), cyanocobaimine 5 mg L^{-1} (3.7 μM), p-aminobenzoic 50 mg L^{-1} (365.0 μM). $FeII(NH_4)_2(SO_4)_2 \cdot 6H_2O$ solution (w:v = 0.1%) comprised the following: $(NH_4)_2SO_4$ 0.00337 g L^{-1} and $FeSO_4 \cdot 7H_2O$ 0.00709 g L^{-1}. The $FeII(NH_4)_2(SO_4)_2 \cdot 6H_2O$ solution has to be prepared in dd.H_2O. 141b medium contained the same ingredients as 141-medium using NaCl 6 g L^{-1}, instead of NaCl 16 g L^{-1}. 141c medium contained the same ingredients as 141-medium including methanol 5 mL L^{-1}.

203 medium Mineral solution 1 (K_2HPO_4 6 g L^{-1}; ultrapure H_2O Milli-Q® system) 37.5 mL L^{-1}, mineral solution 2 (KH_2PO_4 6 g L^{-1}, $(NH_4)_2SO_4$ 6 g L^{-1}, NaCl 12 g L^{-1}, $MgSO_4 \cdot 7H_2O$ 2.4 g L^{-1}, $CaCl_2 \cdot 2H_2O$ 1.6 g L^{-1}; ultrapure H_2O Milli-Q® system) 37.5 mL L^{-1}, $NiCl_2 \cdot 6H_2O$ solution (0.1% w/v) 1 mL L^{-1}, $FeSO_4 \cdot 7H_2O$ solution (0.1% w/v in 0.1 M H_2SO_4) 2 mL L^{-1}. TE solution (TES1) (see medium 141) 10.0 mL, Na_2SO_4 3.4 g, $NaHCO_3$ 2.0 g, Yeast extract (OXOID) 2 g L^{-1}, Trypticase peptone (BD BBL) 2 g L^{-1}, vitamin solution (see medium 141) 10 mL L^{-1}, $Na_2S \cdot 9H_2O$ 0.5 g L^{-1}, L-Cysteine-HCl·H_2O 0.5 g L^{-1}. In case of *Methanothermus sociabilis* yeast extract and trypticase peptone is not needed and was therefore not added to the medium. 203c medium was prepared without the addition of vitamins. 203-c medium was prepared without the vitamins and L-Cysteine-HCl·H_2O.

511 medium NH_4Cl 0.25 g L^{-1}, $K_2HPO_4 \cdot 3H_2O$ 0.07 g L^{-1}, KH_2PO_4 0.09 g L^{-1}, NaCl 11.8 g L^{-1}, $MgSO_4 \cdot 7H_2O$ 1.75 g L^{-1}, $MgCl_2 \cdot 6H_2O$ 4.5 g L^{-1}, Na_2SO_4 0.81 g L^{-1}, $CaCl_2 \cdot 2H_2O$ 0.78 g L^{-1}, KCl 0.3 g L^{-1}. TE solution 511 medium (TES5): 10 mL L^{-1} 141-medium-TES, and marine TE (see below) 10 mL L^{-1}, $(NH_4)_2Fe(SO_4)_2 \cdot 6H_2O$ solution (0.1% w/v) 2 mL L^{-1}, $(NH_4)_2Ni(SO_4)_2$ solution (0.1% w/v) 2mL L^{-1}, $Na_2WO_4 \cdot 2H_2O$ solution (0.1% w/v) 2 mL L^{-1}, $NaHCO_3$ 1 g L^{-1}, vitamins solution (see medium 141) 10 mL L^{-1}, $Na_2S \cdot 9H_2O$ 0.5 g L^{-1}. Marine TE solution: NaBr 4 g L^{-1}, $SrCl_2 \cdot 6H_2O$ 1.8 g L^{-1}, H_3BO_3 1.3 g L^{-1}, KI 1.25 mg L^{-1}, Na-silicate 100 mg L^{-1}, NaF 60 mg L^{-1}, KNO_3 40 mg L^{-1}, $Na_2HPO_4 \cdot 3H_2O$ 0.25 mg L^{-1}. 511-v medium was prepared without vitamins.

SAB medium (modified): $MgSO_4 \cdot 7H_2O$ 0.8 g L^{-1}, KH_2PO_4 0.5 g L^{-1}, K_2HPO_4 0.5 g L^{-1}, $CaCl_2 \cdot 6H_2O$ 0.05 g L^{-1}, NaCl 1.5 g L^{-1}, NH_4Cl 1 g L^{-1}, $Na_2S \cdot 9H_2O$ 0.3 g L^{-1}, sodium acetate 1 g L^{-1}, yeast extract 2 g L^{-1}, 5mM valeric acid, 5 mM isovaleric acid, 5 mM 2-methylbutyric acid, 6 mM isobutyric acid, 5 mM 2-methyl valeric acid, 1000x TE solution 1 mL L^{-1}. The

SAB-TE solution (TES2) contained the following: nitrilotriacetic acid 1.5 g L^{-1}, $NiCl_2 \cdot 6H_2O$ 15 g L^{-1}, KCl 0.5 g L^{-1}, $MnSO_4 \cdot 7H_2O$ 6 g L^{-1}, $ZnSO_4 \cdot 7H_2O$ 1 g L^{-1}, $CuSO_4$ 1.278 g L^{-1}, $KAl(SO_4)_2 \cdot 12H_2O$ 2 mg L^{-1}, H_3BO_3 70 mg L^{-1}, $CoSO_4 \cdot 7H_2O$ 40 mg L^{-1}, $Na_2MoO_3 \cdot H_2O$ 5 g L^{-1}, $Na_2SeO_3 \cdot 5H_2O$ 30 mg L^{-1}, $Na_2WO_4 \cdot 2H_2O$ 40 mg L^{-1}, $FeSO_4 \cdot 7H_2O$ 9 g L^{-1}. After anaerobization and autoclaving the medium, L-Cysteine-HCl·H_2O 0.5 g L^{-1}, 4 M methanol 20 mL L^{-1}, 8 M sodium format 20 mL L^{-1}, 10% $NaHCO_3$ 20 mL L^{-1}, 2% $Na_2S \cdot 9H_2O$ 20 mL L^{-1} and vitamin solution 15 mL L^{-1} (Wolf´s vitamin solution) must be added to the serum bottles. The pH in the serum bottles was adjusted to 7.5 with 10 M KOH.

Medium 6 NH_4Cl 1 g L^{-1}, $MgCl_2 \cdot 6H_2O$ 1 g L^{-1}, $CaCl_2 \cdot 2H_2O$ 0.4 g L^{-1}, $KH_2PO_4 \cdot 2H_2O$ 0.6325 g L^{-1}, $NaHCO_3$ 12 g L^{-1}, $Na_2S \cdot 9H_2O$ 0.9092 g L^{-1}. TE solution 10 mL L^{-1}, vitamin solution[1] 10 mL L^{-1}. TE solution (TES6)[2]: nitrilotriacetic acid 1.5 g L^{-1}, dissolve nitrilotriacetic acid with KOH to pH 6.5; then proceed with mineral addition: $MgSO_4 \cdot 7H_2O$ 3 g L^{-1}, $MnCl_2 \cdot 4H_2O$ 0.529 g L^{-1}, NaCl 1 g L^{-1}, $FeSO_4 \cdot 7H_2O$ 0.1 g L^{-1}, $CoCl_2 \cdot 6H_2O$ 0.1833 g L^{-1}, $CaCl_2 \cdot 2H_2O$ 0.1 g L^{-1}, $ZnSO_4 \cdot 7H_2O$ 0.1781 g L^{-1}, $CuSO_4$ 0.0063 g L^{-1}, $AlK(SO_4)_2$ 0.01 g L^{-1}, $AlK(SO_4)_2 \cdot 12H_2O$ 0.0184 g L^{-1}, H_3BO_3 0.01 g L^{-1}, $Na_2MoO_4 \cdot 2H_2O$ 0.01 g L^{-1}(pH to 7.0 with KOH).

McN medium for Methanococci contained: KCl 0.335 g L^{-1}, $MgCl_2 \cdot 6H_2O$ 2.75 g L^{-1}, $MgSO_4 \cdot 7H_2O$ 3.45 g L^{-1}, NH_4Cl 0.5 g L^{-1}, $CaCl_2 \cdot 2H_2O$ 0.14 g L^{-1}, K_2HPO_4 1.4 g L^{-1} and NaCl 21.975 g L^{-1}. TE solution (TES3) 10 mL L^{-1} (MM medium TE solution), iron stock solution 5 mL L^{-1}, After anaerobization and autoclaving the medium, $Na_2S \cdot 9H_2O$ 0.5 g L^{-1} must be added. $Fe(NH_4)_2(SO_4)_2$ solution for MCN medium: 2 g, $Fe(NH_4)_2(SO_4)_2 \cdot 6H_2O$ and 100 µL conc. HCl must be mixed and filled up to 1 L with ultrapure H_2O Milli-Q®.

282c 0, 282c 18, or 282c 30 medium (282 medium with cysteine and 0 g, 18 g, or 30 g NaCl): K_2HPO_4 0.14 g L^{-1}, $CaCl_2 \cdot 2H_2O$ 0.14 g L^{-1}, NH_4Cl 0.25 g L^{-1}, $MgSO_4 \cdot 7H_2O$ 3.4 g L^{-1}, $MgCl_2 \cdot 6H_2O$ 4.1 g L^{-1}, KCl 0.33 g L^{-1}, $NiCl_2 \cdot 6H_2O$ solution (0.1% w/v) 0.5 mL, NaCl 0 g L^{-1} (*M. palustre*), 18 g L^{-1} (*M. villosus*) or 30 g L^{-1} (*M. vulcanius*, *M. jannaschii*), $Fe(NH_4)_2(SO_4)_2 \cdot 6H_2O$ 0.01 g L^{-1}, TE solution (TES1) (DSMZ medium 141) 10 mL L^{-1}. After anaerobization and autoclaving the medium, $NaHCO_3$ 1 g L^{-1}, L-Cysteine-HCl·H_2O 0.5 g L^{-1} and $Na_2S \cdot 9H_2O$ 0.5 g L^{-1}have to be added. 282-c 30 was prepared without the addition of L-Cysteine-HCl·H_2O.

282c 18_E medium K_2HPO_4 0.14 g L^{-1}, $CaCl_2 \cdot 2H_2O$ 0.14 g L^{-1}, NH_4Cl 0.25 g L^{-1}, $MgSO_4 \cdot 7H_2O$ 3.4 g L^{-1}, $MgCl_2 \cdot 6H_2O$ 4.1 g L^{-1}, KCl 0.33 g L^{-1}, $NiCl_2 \cdot 6H_2O$ 0.5 mg L^{-1}, $Na_2SeO_3 \cdot 5H_2O$ 0.5 mg L^{-1}, NaCl 18 g L^{-1} (*M. villosus*) or 30 g L^{-1} (*M. vulcanius*, *M. jannaschii*), $FeSO_4 \cdot 7H_2O$ 7 mg L^{-1}, TE solution (TES1) (DSMZ medium 141) 10 mL L^{-1}. After anaerobization and autoclaving the medium, $NaHCO_3$ 1 g L^{-1}, L-Cysteine-HCl·H_2O 0.5 g L^{-1}, $Na_2S \cdot 9H_2O$ 0.5 g L^{-1} must be added. TE solution for 282 medium contained the following: nitrilotriacetic acid 1.5 g L^{-1}, $MgSO_4 \cdot 7H_2O$ 3 g L^{-1}, $MnCl_2 \cdot 4H_2O$ 0.585 g L^{-1}, NaCl 1 g L^{-1}, $FeSO_4 \cdot 7H_2O$ 0.1 g L^{-1}, $CoSO_4 \cdot 7H_2O$ 0.18 g L^{-1}, $CaCl_2 \cdot 2H_2O$ 0.1 g L^{-1}, $ZnSO_4 \cdot 7H_2O$ 0.18 g L^{-1}, $CuSO_4$ 0.006 g L^{-1}, $KAI(SO_4)_2 \cdot 12H_2O$ 0.02 g L^{-1}, H_3BO_3 0.01 g L^{-1}, $Na_2MoO_4 \cdot 2H_2O$ 0.01 g L^{-1}, $NiCl_2 \cdot 6H_2O$ 0.03 g L^{-1}, $Na_2SeO_3 \cdot 5H_2O$ 0.3 mg L^{-1}, $Na_2WO_4 \cdot 2H_2O$ 0.4 mg L^{-1}.

Methanothermobacter marburgensis **medium (MM)** contained the following compounds: NH_4Cl 2.1 g L^{-1}, KH_2PO_4 6.8 g L^{-1}, Na_2CO_3 3.6 g L^{-1}, 100x TE 10 mL L^{-1}. After anaerobization and autoclaving the medium, 0.5 M $Na_2S{\cdot}9H_2O$ 2 mL L^{-1} must be added. 100x TE solution (TES3) contained: Titriplex I 9 g L^{-1}, add 800 mL ultrapure H_2O Milli-Q® system and adjust the pH to 6.5 with 5M NaOH solution, then add: $MgCl_2{\cdot}6H_2O$ 4 g L^{-1}, $FeCl_2{\cdot}4H_2O$ 1 g L^{-1}, $CoCl_2{\cdot}6H_2O$ 20 mg L^{-1}, $NiCl_2{\cdot}6H_2O$ 120 mg L^{-1}, $NaMoO_4{\cdot}2H_2O$ 20 mg L^{-1}, put to pH= 7.0 with 1 M NaOH. MM15 and MM30 contained additionally 15 or 30 g L^{-1} NaCl. MM15c and MM30c contained additionally L-Cysteine-HCl·H_2O 0.5 g L^{-1}. MM15v contained additionally 10 mL L^{-1} of vitamins (medium 141).

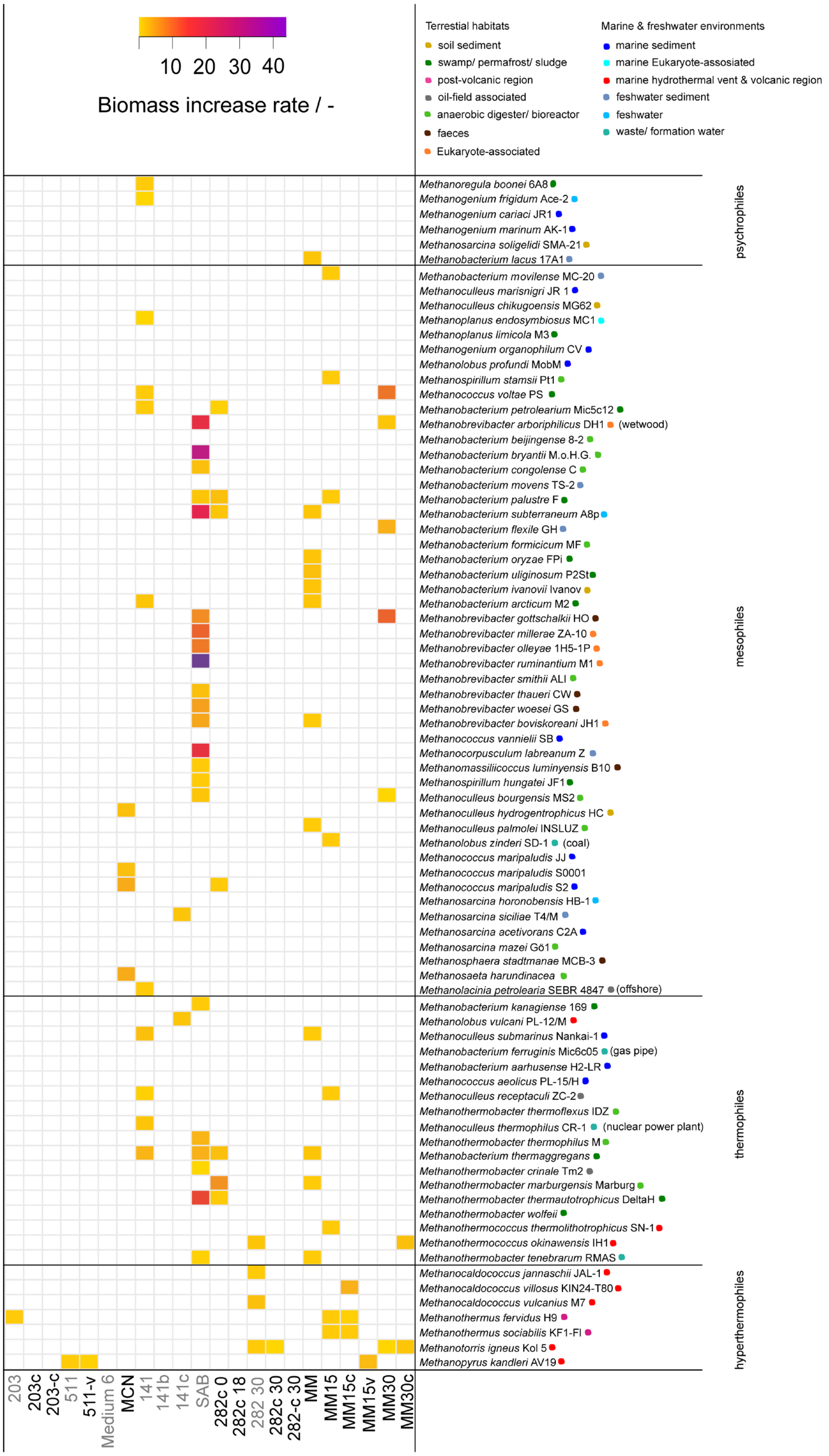

10 20 30 40
Biomass increase rate / -
Terrestial habitats
soil sediment
swamp/ permafrost/ sludge
post-volcanic region
oil-field associated
anaerobic digester/ bioreactor
faeces
Eukaryote-associated
Marine & freshwater environments
marine sediment
marine Eukaryote-associated
marine hydrothermal vent & volcanic region
feshwater sediment
feshwater
waste/ formation water
Methanoregula boonei 6A8
Methanogenium frigidum Ace-2
Methanogenium cariaci JR1
Methanogenium marinum AK-1
Methanosarcina soligelidi SMA-21
Methanobacterium lacus 17A1
Methanobacterium movilense MC-20
Methanoculleus marisnigri JR 1
Methanoculleus chikugoensis MG62
Methanoplanus endosymbiosus MC1
Methanoplanus limicola M3
Methanogenium organophilum CV
Methanolobus profundi MobM
Methanospirillum stamsii Pt1
Methanococcus voltae PS
Methanobacterium petrolearium Mic5c12
Methanobrevibacter arboriphilicus DH1 (wetwood)
Methanobacterium beijingense 8-2
Methanobacterium bryantii M.o.H.G.
Methanobacterium congolense C
Methanobacterium movens TS-2
Methanobacterium palustre F
Methanobacterium subterraneum A8p
Methanobacterium flexile GH
Methanobacterium formicicum MF
Methanobacterium oryzae FPi
Methanobacterium uliginosum P2St
Methanobacterium ivanovii Ivanov
Methanobacterium arcticum M2
Methanobrevibacter gottschalkii HO
Methanobrevibacter millerae ZA-10
Methanobrevibacter olleyae 1H5-1P
Methanobrevibacter ruminantium M1
Methanobrevibacter smithii ALI
Methanobrevibacter thaueri CW
Methanobrevibacter woesei GS
Methanobrevibacter boviskoreani JH1
Methanococcus vannielii SB
Methanocorpusculum labreanum Z
Methanomassiliicoccus luminyensis B10
Methanospirillum hungatei JF1
Methanoculleus bourgensis MS2
Methanoculleus hydrogentrophicus HC
Methanoculleus palmolei INSLUZ
Methanolobus zinderi SD-1 (coal)
Methanococcus maripaludis JJ
Methanococcus maripaludis S0001
Methanococcus maripaludis S2
Methanosarcina horonobensis HB-1
Methanosarcina siciliae T4/M
Methanosarcina acetivorans C2A
Methanosarcina mazei Gö1
Methanosphaera stadtmanae MCB-3
Methanosaeta harundinacea
Methanolacinia petrolearia SEBR 4847 (offshore)
Methanobacterium kanagiense 169
Methanolobus vulcani PL-12/M
Methanoculleus submarinus Nankai-1
Methanobacterium ferruginis Mic6c05 (gas pipe)
Methanobacterium aarhusense H2-LR
Methanococcus aeolicus PL-15/H
Methanoculleus receptaculi ZC-2
Methanothermobacter thermoflexus IDZ
Methanoculleus thermophilus CR-1 (nuclear power plant)
Methanothermobacter thermophilus M
Methanobacterium thermaggregans
Methanothermobacter crinale Tm2
Methanothermobacter marburgensis Marburg
Methanothermobacter thermautotrophicus DeltaH
Methanothermobacter wolfeii
Methanothermococcus thermolithotrophicus SN-1
Methanothermococcus okinawensis IH1
Methanothermobacter tenebrarum RMAS
Methanocaldococcus jannaschii JAL-1
Methanocaldococcus villosus KIN24-T80
Methanocaldococcus vulcanius M7
Methanothermus fervidus H9
Methanothermus sociabilis KF1-F1
Methanotorris igneus Kol 5
Methanopyrus kandleri AV19
psychrophiles
mesophiles
thermophiles
hyperthermophiles
203
203c
203-c
511
511-v
Medium 6
MCN
141
141b
141c
SAB
282c 0
282c 18
282 30
282c 30
282-c 30
MM
MM15
MM15c
MM15v
MM30
MM30c

Fig. S1 Results of the multivariate pre-screen of 80 methanogens in defined and complex media respectively to the biomass increase rate. Experiments were performed in closed batch cultivation systems at 2 bar (120mL flasks, 50 mL medium). On the y-axis, methanogens were arranged as groups according to their temperature optimum in psychrophiles, mesophiles, thermophiles or hyperthermophiles. Methanogens are listed with ascending strain specific temperature optimum from top to bottom. Coloured points next to the strain designation on the y-axis indicate the isolation site of the tested methanogen (terrestrial habitats: golden brown - soil sediment, dark green - swamp/permafrost/sludge, pink - post-volcanic region, grey - oil-field associated, light green - anaerobic digester/ bioreactor, brown - faeces, orange - eukaryote-associated; marine and freshwater environments: bright blue - marine sediment, turquoise - marine eukaryote-associated, red - marine hydrothermal vent and volcanic region, grey blue - freshwater sediment, sky blue - freshwater, green blue - waste/formation water). In total, 22 defined and complex media were tested, but not every strain could be or was cultivated on every medium. Defined and complex media are shown on the x-axis in black and grey fonts, respectively. For each closed batch cultivation, three biological replicates (in some cases, two biological replicates) plus one negative control were used.

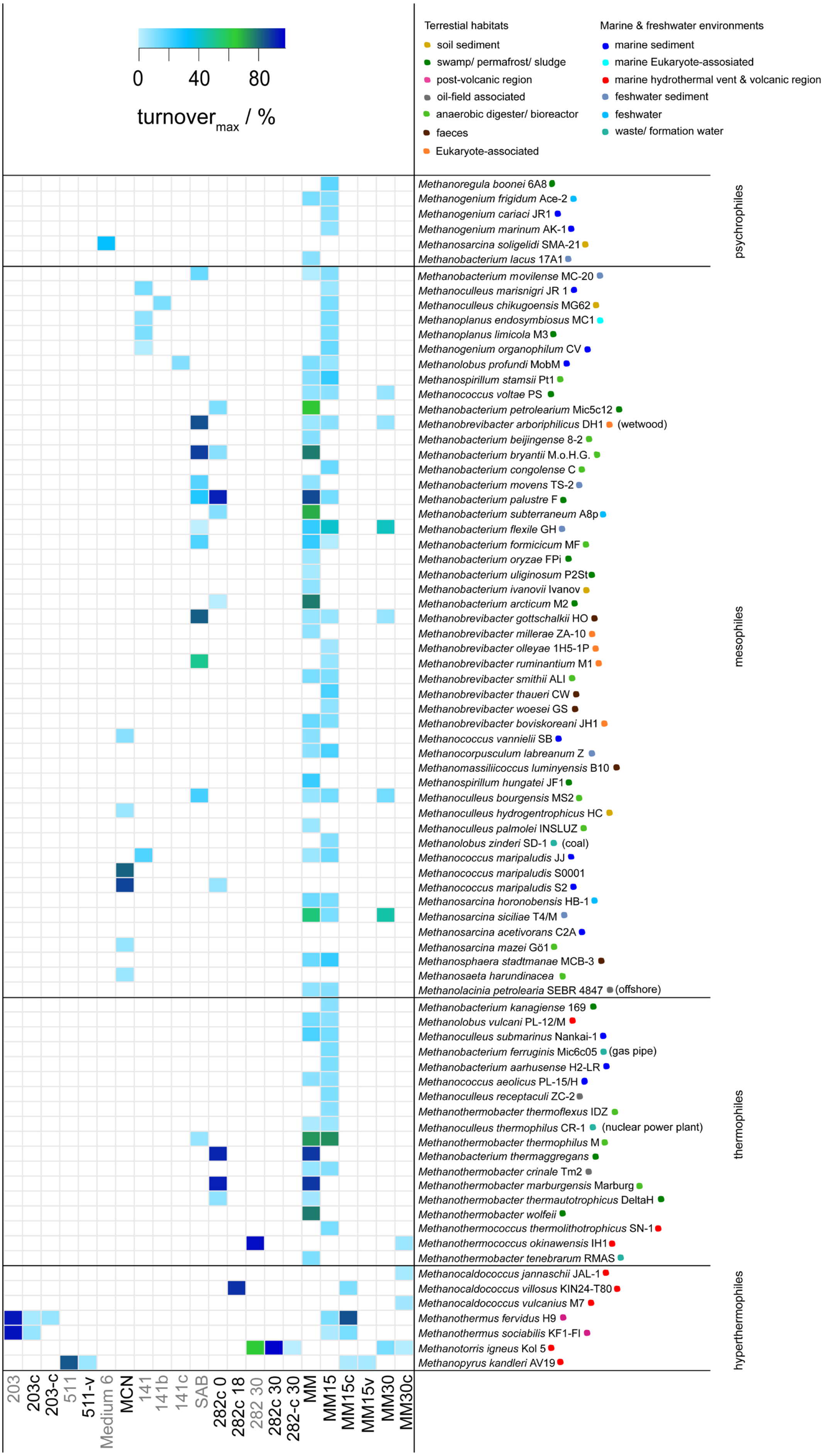

0
40
80
turnover$_{max}$ / %
Terrestial habitats
soil sediment
swamp/ permafrost/ sludge
post-volcanic region
oil-field associated
anaerobic digester/ bioreactor
faeces
Eukaryote-associated
Marine & freshwater environments
marine sediment
marine Eukaryote-assosiated
marine hydrothermal vent & volcanic region
feshwater sediment
feshwater
waste/ formation water
psychrophiles
Methanoregula boonei 6A8
Methanogenium frigidum Ace-2
Methanogenium cariaci JR1
Methanogenium marinum AK-1
Methanosarcina soligelidi SMA-21
Methanobacterium lacus 17A1
mesophiles
Methanobacterium movilense MC-20
Methanoculleus marisnigri JR 1
Methanoculleus chikugoensis MG62
Methanoplanus endosymbiosus MC1
Methanoplanus limicola M3
Methanogenium organophilum CV
Methanolobus profundi MobM
Methanospirillum stamsii Pt1
Methanococcus voltae PS
Methanobacterium petrolearium Mic5c12
Methanobrevibacter arboriphilicus DH1 (wetwood)
Methanobacterium beijingense 8-2
Methanobacterium bryantii M.o.H.G.
Methanobacterium congolense C
Methanobacterium movens TS-2
Methanobacterium palustre F
Methanobacterium subterraneum A8p
Methanobacterium flexile GH
Methanobacterium formicicum MF
Methanobacterium oryzae FPi
Methanobacterium uliginosum P2St
Methanobacterium ivanovii Ivanov
Methanobacterium arcticum M2
Methanobrevibacter gottschalkii HO
Methanobrevibacter millerae ZA-10
Methanobrevibacter olleyae 1H5-1P
Methanobrevibacter ruminantium M1
Methanobrevibacter smithii ALI
Methanobrevibacter thaueri CW
Methanobrevibacter woesei GS
Methanobrevibacter boviskoreani JH1
Methanococcus vannielii SB
Methanocorpusculum labreanum Z
Methanomassiliicoccus luminyensis B10
Methanospirillum hungatei JF1
Methanoculleus bourgensis MS2
Methanoculleus hydrogentrophicus HC
Methanoculleus palmolei INSLUZ
Methanolobus zinderi SD-1 (coal)
Methanococcus maripaludis JJ
Methanococcus maripaludis S0001
Methanococcus maripaludis S2
Methanosarcina horonobensis HB-1
Methanosarcina siciliae T4/M
Methanosarcina acetivorans C2A
Methanosarcina mazei Gö1
Methanosphaera stadtmanae MCB-3
Methanosaeta harundinacea
Methanolacinia petrolearia SEBR 4847 (offshore)
thermophiles
Methanobacterium kanagiense 169
Methanolobus vulcani PL-12/M
Methanoculleus submarinus Nankai-1
Methanobacterium ferruginis Mic6c05 (gas pipe)
Methanobacterium aarhusense H2-LR
Methanococcus aeolicus PL-15/H
Methanoculleus receptaculi ZC-2
Methanothermobacter thermoflexus IDZ
Methanoculleus thermophilus CR-1 (nuclear power plant)
Methanothermobacter thermophilus M
Methanobacterium thermaggregans
Methanothermobacter crinale Tm2
Methanothermobacter marburgensis Marburg
Methanothermobacter thermautotrophicus DeltaH
Methanothermobacter wolfeii
Methanothermococcus thermolithotrophicus SN-1
Methanothermococcus okinawensis IH1
Methanothermobacter tenebrarum RMAS
hyperthermophiles
Methanocaldococcus jannaschii JAL-1
Methanocaldococcus villosus KIN24-T80
Methanocaldococcus vulcanius M7
Methanothermus fervidus H9
Methanothermus sociabilis KF1-FI
Methanotorris igneus Kol 5
Methanopyrus kandleri AV19
203
203c
203-c
511
511-v
Medium 6
MCN
141
141b
141c
SAB
282c 0
282c 18
282 30
282c 30
282-c 30
MM
MM15
MM15c
MM15v
MM30
MM30c

Fig. S2 Results of the maximum gas conversion ($turnover_{max}$ / %) of 80 methanogens in defined and complex media during the multivariate pre-screening. Experiments were performed in closed batch cultivation systems at 2 bar (120mL flasks, 50 mL medium). On the y-axis, methanogens were arranged as groups according to their temperature optimum in psychrophiles, mesophiles, thermophiles, or hyperthermophiles. Methanogens are listed with ascending strain specific temperature optimum from top to bottom. Coloured points next to the strain designation on the y-axis indicate the isolation site of the tested methanogen (terrestrial habitats: golden brown - soil sediment, dark green - swamp/permafrost/sludge, pink - post-volcanic region, grey - oil-field associated, light green - anaerobic digester/ bioreactor, brown - faeces, orange - eukaryote-associated; marine and freshwater environments: bright blue - marine sediment, turquoise - marine eukaryote-associated, red - marine hydrothermal vent and volcanic region, grey blue - freshwater sediment, sky blue - freshwater, green blue - waste/formation water). In total, 22 defined and complex media were tested, but not every strain could be or was cultivated on every medium. Defined and complex media are shown on the x-axis in black and grey fonts, respectively. For each closed batch cultivation, three biological replicates (in some cases, two biological replicates) plus one negative control were used.

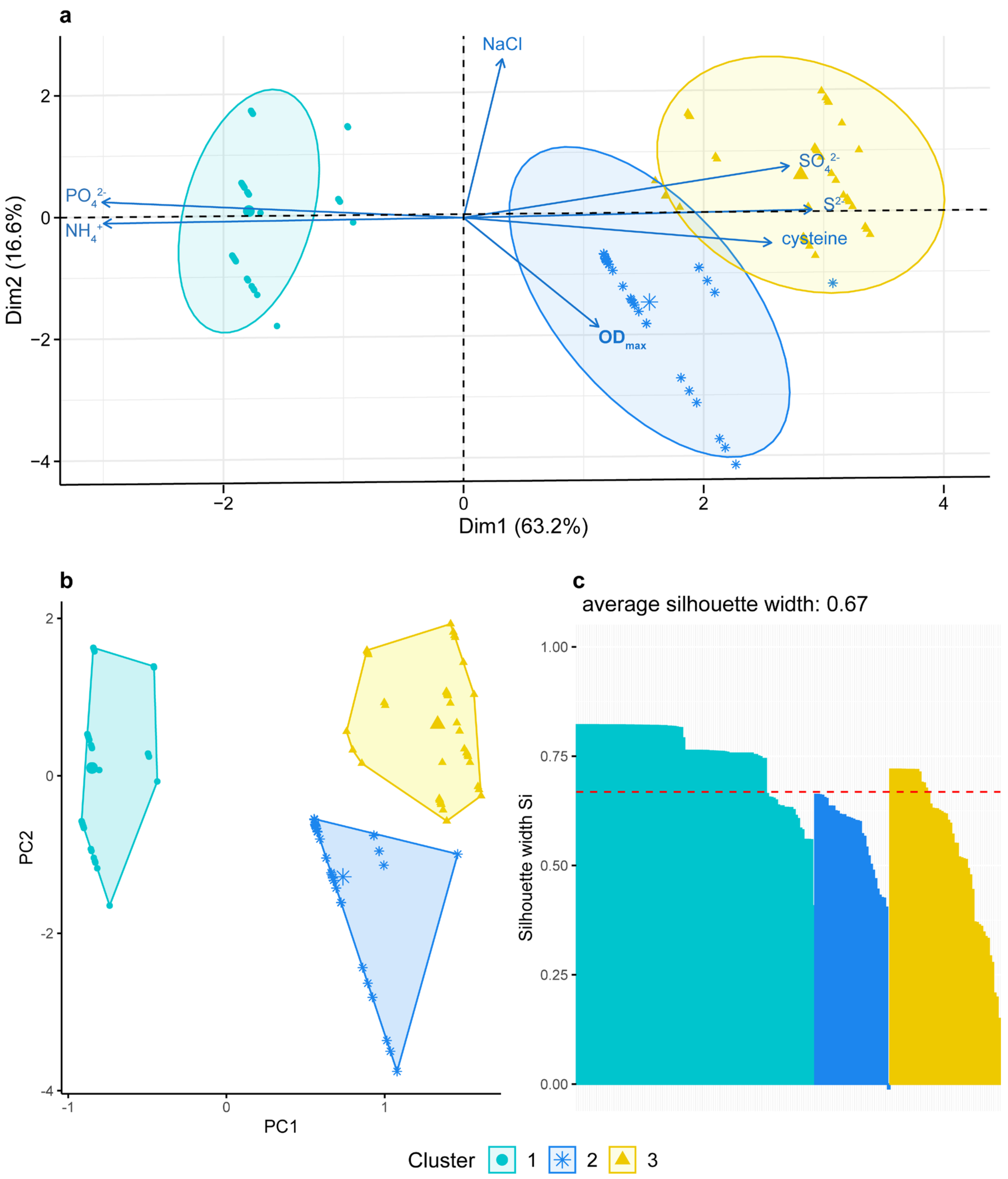

Fig. S3 k-means cluster analysis of the nutrimental demand and associated growth (OD_{max}) of tested methanogens. Fig. S3a shows a biplot including the variance of the variables and the corresponding clusters. Fig. S3b illustrates the k-means clusters after the PCA. For the k-means clustering two principle components were used. Fig. S3c shows a silhouette plot (quality of clustering) of the cluster.

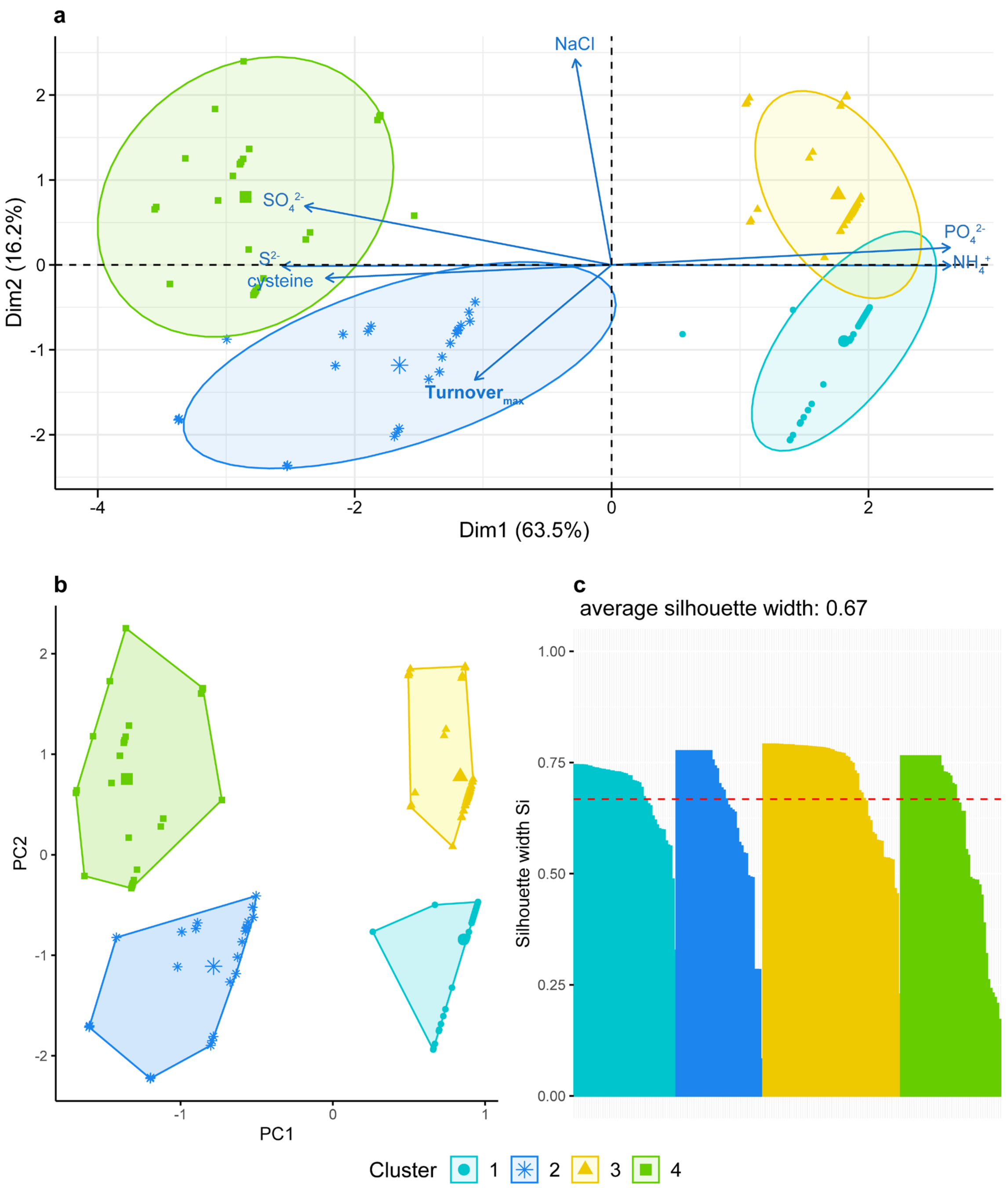

Fig. S4 k-means cluster analysis of the nutrimental demand and associated turnover of the substrates ($turnover_{max}$) of tested methanogens. Fig. S4a shows a biplot including the variance of the variables and the corresponding clusters. Fig. S4b illustrates the k-means clusters after the PCA. For the k-means clustering two principle components were used. Fig. S4c shows a silhouette plot (quality of clustering) of the cluster.

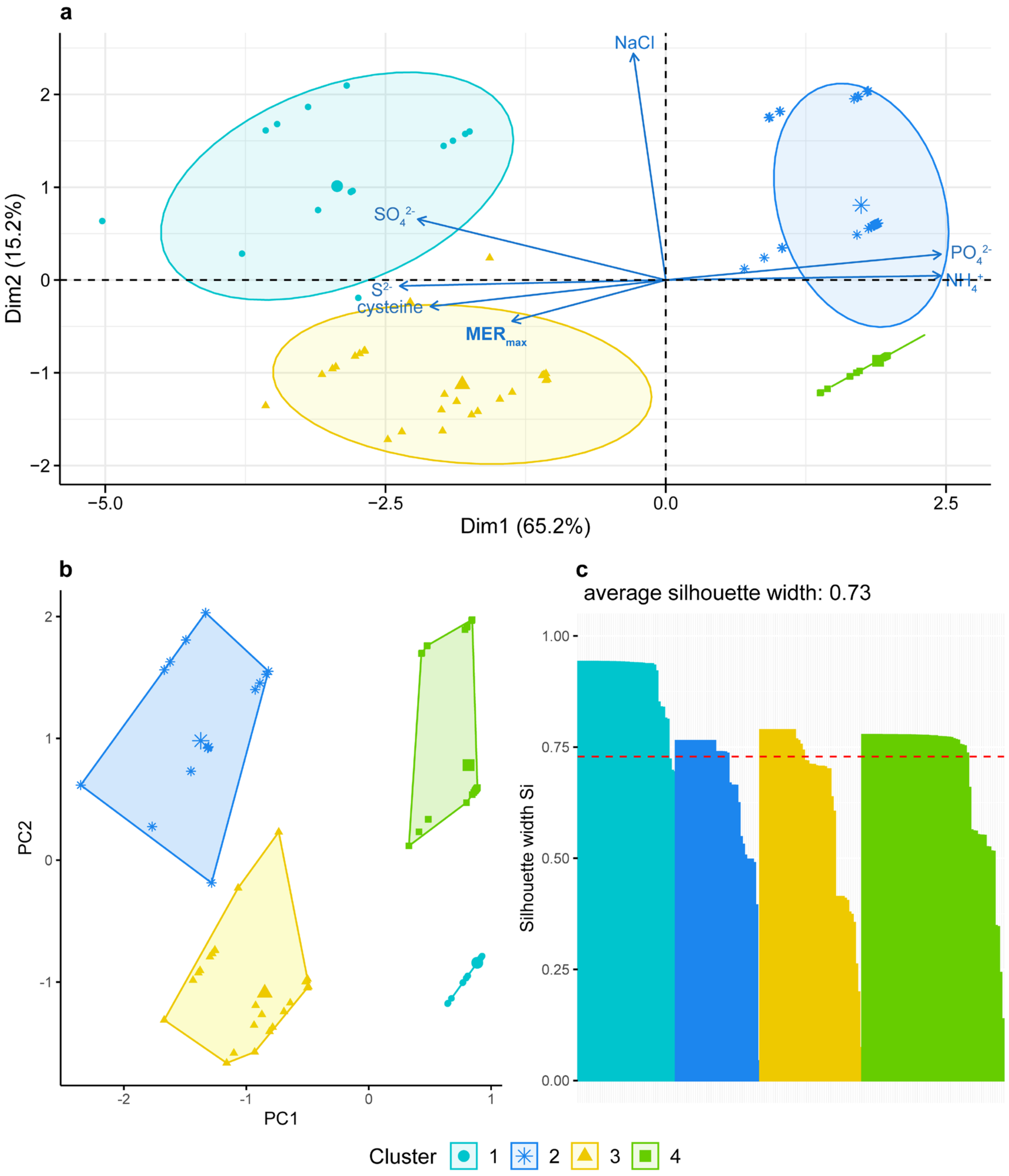

Fig. S5 k-means cluster analysis of the nutrimental demand and associated volumetic CH_4 production rate (MER_{max}) of tested methanogens. Fig. S5a shows a biplot including the variance of the varibles and the corresponding clusters. Fig. S5b illustrates the k-means clusters after the PCA. For the k-means clustering two principle components were used. Fig. S5c shows a silhouette plot (quality of clustering) of the cluster.

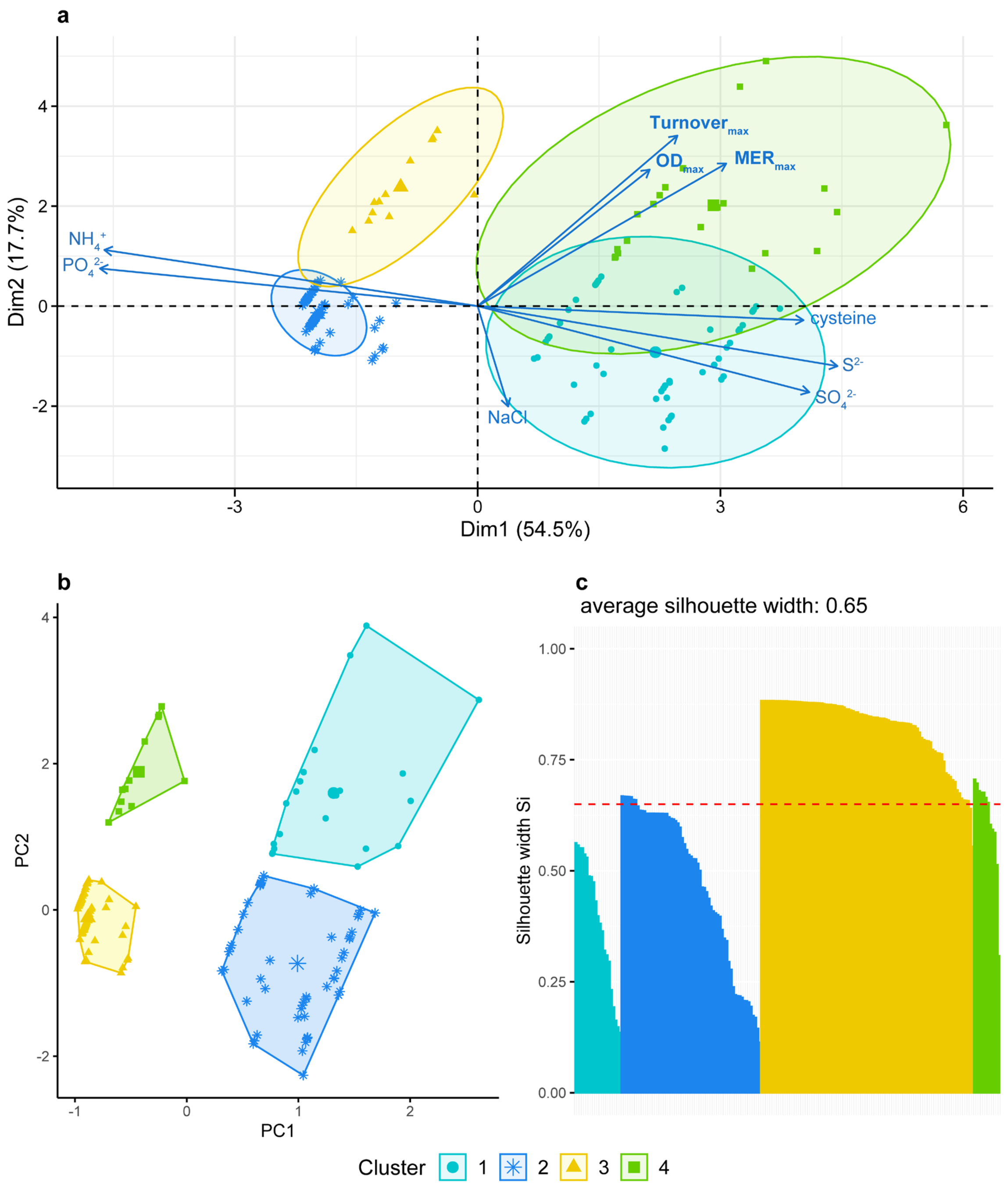

Fig. S6 k-means cluster analysis of the nutrimental demand and associated gowth (OD_{max}), turnover of substrates ($turnover_{max}$), and volumetic CH_4 production rate (MER_{max}) of tested methanogens. Fig. S6a shows a biplot including the variance of the varibles and the corresponding clusters. Fig. S6b illustrates the k-means clusters after the PCA. For the k-means clustering two principle components were used. Fig. S6c shows a silhouette plot (quality of clustering) of the cluster.

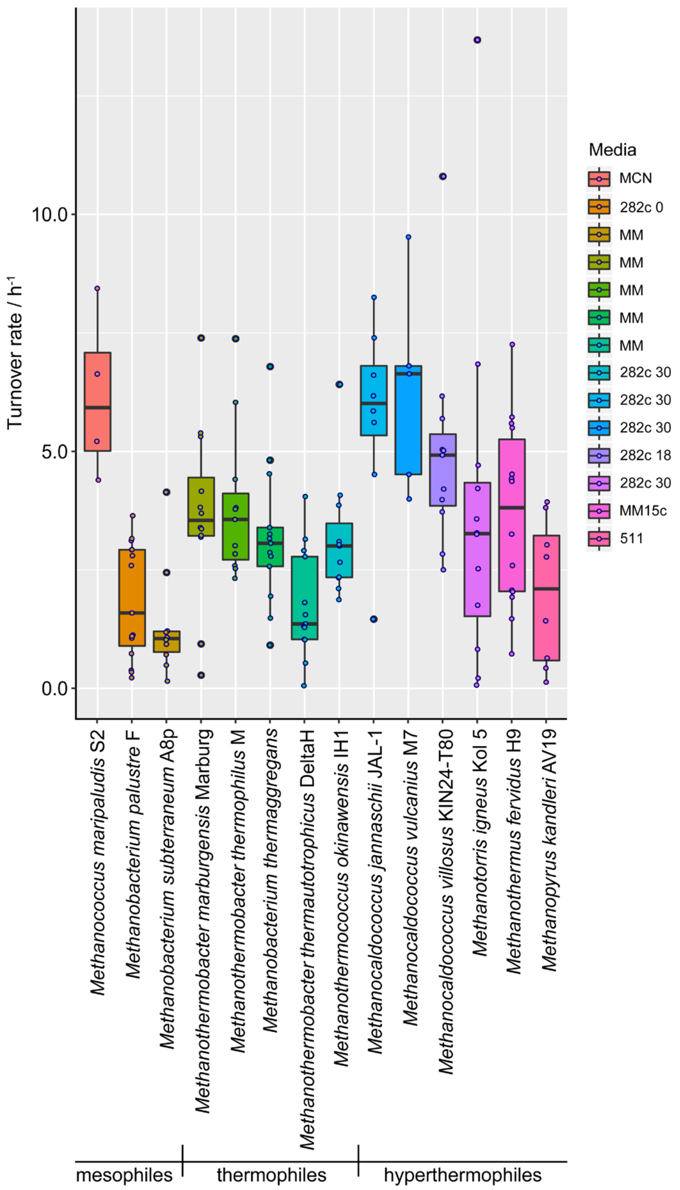

Fig. S7 Repetitive closed-batch cultivation using high frequency gassing (HFG) for prioritized methanogens at 2 bar in defined medium. All experiments were performed in quadruplicates including a negative control. The turnover rate / h^{-1} is shown. Three temperature blocks are distinguished and highlighted from left to right: mesophilic methanogens grown at 37°C (*Methanococcus maripaludis* S2, *Methanobacterium palustre* F, *Methanobacterium subterraneum* A8p); middle block: five thermophilic strains grown at 65°C (*Methanothermobacter marburgensis* Marburg, *Methanothermobacter thermophilus* M, *Methanobacterium thermaggregans*, *Methanothermobacter thermautotrophicus* DeltaH, *Methanothermococcus okinawensis* IH1); right block: six hyperthermophilic methanogens (*Methanocaldococcus jannaschii* JAL-1 (80°C), *Methanocaldococcus vulcanius* M7 (80°C), *Methanocaldococcus villosus* KIN24-T80 (80°C), *Methanotorris igneus* Kol 5 (85°C), *Methanothermus fervidus* H9 (80°C), *Methanopyrus kandleri* AV19 (98°C)).

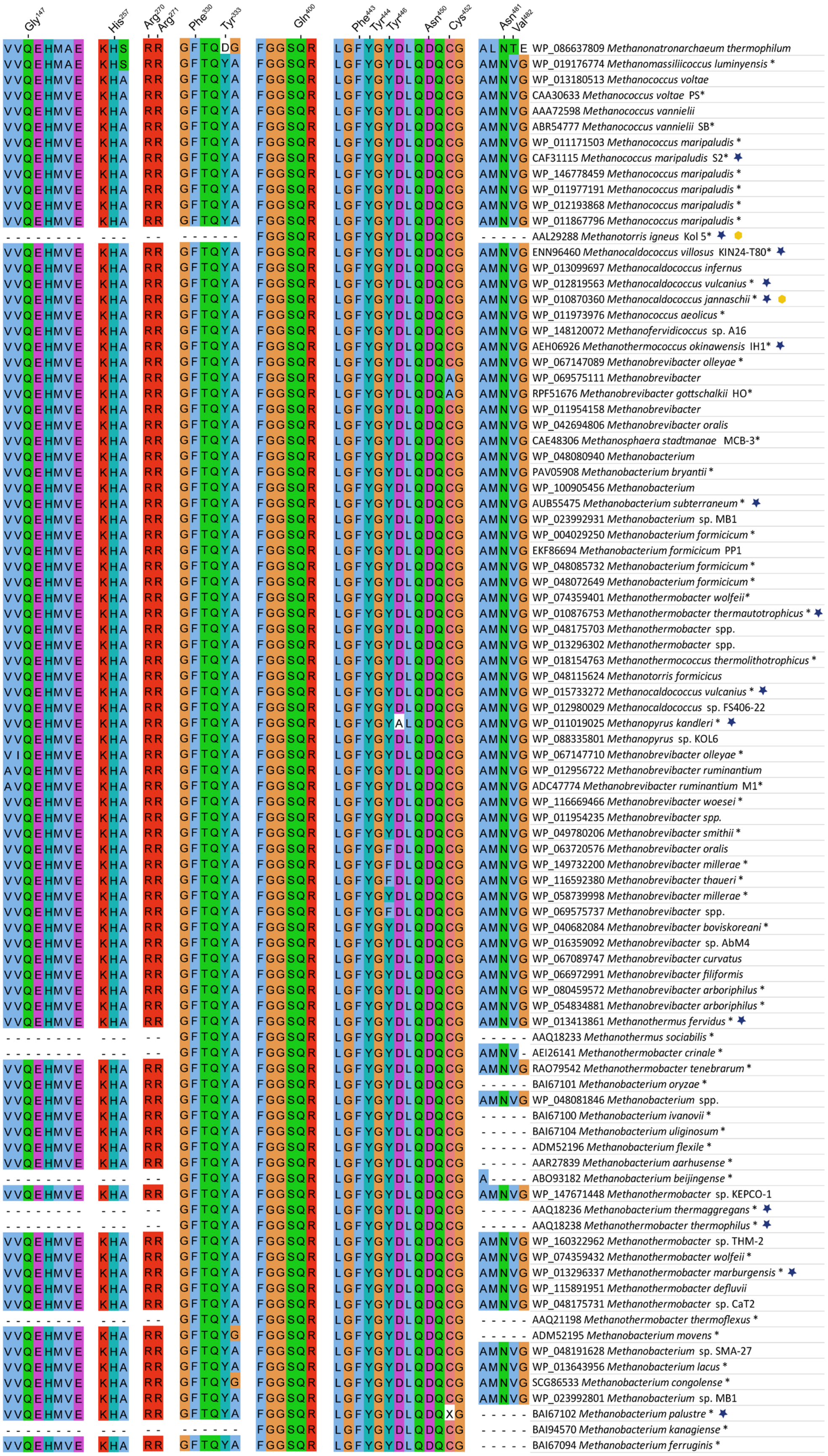

Gly447
His357
Arg270
Arg271
Phe330
Tyr333
Gln400
Phe443
Tyr444
Tyr446
Asn450
Cys452
Asn481
Val482
WP_086637809 Methanonatronarchaeum thermophilum
WP_019176774 Methanomassiliicoccus luminyensis *
WP_013180513 Methanococcus voltae
CAA30633 Methanococcus voltae PS*
AAA72598 Methanococcus vannielii
ABR54777 Methanococcus vannielii SB*
WP_011171503 Methanococcus maripaludis *
CAF31115 Methanococcus maripaludis S2*
WP_146778459 Methanococcus maripaludis *
WP_011977191 Methanococcus maripaludis *
WP_012193868 Methanococcus maripaludis *
WP_011867796 Methanococcus maripaludis *
AAL29288 Methanotorris igneus Kol 5*
ENN96460 Methanocaldococcus villosus KIN24-T80*
WP_013099697 Methanocaldococcus infernus
WP_012819563 Methanocaldococcus vulcanius *
WP_010870360 Methanocaldococcus jannaschii *
WP_011973976 Methanococcus aeolicus *
WP_148120072 Methanofervidicoccus sp. A16
AEH06926 Methanothermococcus okinawensis IH1*
WP_067147089 Methanobrevibacter olleyae *
WP_069575111 Methanobrevibacter
RPF51676 Methanobrevibacter gottschalkii HO*
WP_011954158 Methanobrevibacter
WP_042694806 Methanobrevibacter oralis
CAE48306 Methanosphaera stadtmanae MCB-3*
WP_048080940 Methanobacterium
PAV05908 Methanobacterium bryantii *
WP_100905456 Methanobacterium
AUB55475 Methanobacterium subterraneum *
WP_023992931 Methanobacterium sp. MB1
WP_004029250 Methanobacterium formicicum *
EKF86694 Methanobacterium formicicum PP1
WP_048085732 Methanobacterium formicicum *
WP_048072649 Methanobacterium formicicum *
WP_074359401 Methanothermobacter wolfeii*
WP_010876753 Methanothermobacter thermautotrophicus *
WP_048175703 Methanothermobacter spp.
WP_013296302 Methanothermobacter spp.
WP_018154763 Methanothermococcus thermolithotrophicus *
WP_048115624 Methanotorris formicicus
WP_015733272 Methanocaldococcus vulcanius *
WP_012980029 Methanocaldococcus sp. FS406-22
WP_011019025 Methanopyrus kandleri *
WP_088335801 Methanopyrus sp. KOL6
WP_067147710 Methanobrevibacter olleyae *
WP_012956722 Methanobrevibacter ruminantium
ADC47774 Methanobrevibacter ruminantium M1*
WP_116669466 Methanobrevibacter woesei *
WP_011954235 Methanobrevibacter spp.
WP_049780206 Methanobrevibacter smithii *
WP_063720576 Methanobrevibacter oralis
WP_149732200 Methanobrevibacter millerae *
WP_116592380 Methanobrevibacter thaueri *
WP_058739998 Methanobrevibacter millerae *
WP_069575737 Methanobrevibacter spp.
WP_040682084 Methanobrevibacter boviskoreani *
WP_016359092 Methanobrevibacter sp. AbM4
WP_067089747 Methanobrevibacter curvatus
WP_066972991 Methanobrevibacter filiformis
WP_080459572 Methanobrevibacter arboriphilus *
WP_054834881 Methanobrevibacter arboriphilus *
WP_013413861 Methanothermus fervidus *
AAQ18233 Methanothermus sociabilis *
AEI26141 Methanothermobacter crinale *
RAO79542 Methanothermobacter tenebrarum *
BAI67101 Methanobacterium oryzae *
WP_048081846 Methanobacterium spp.
BAI67100 Methanobacterium ivanovii *
BAI67104 Methanobacterium uliginosum *
ADM52196 Methanobacterium flexile *
AAR27839 Methanobacterium aarhusense *
ABO93182 Methanobacterium beijingense *
WP_147671448 Methanothermobacter sp. KEPCO-1
AAQ18236 Methanobacterium thermaggregans *
AAQ18238 Methanothermobacter thermophilus *
WP_160322962 Methanothermobacter sp. THM-2
WP_074359432 Methanothermobacter wolfeii *
WP_013296337 Methanothermobacter marburgensis *
WP_115891951 Methanothermobacter defluvii
WP_048175731 Methanothermobacter sp. CaT2
AAQ21198 Methanothermobacter thermoflexus *
ADM52195 Methanobacterium movens *
WP_048191628 Methanobacterium sp. SMA-27
WP_013643956 Methanobacterium lacus *
SCG86533 Methanobacterium congolense *
WP_023992801 Methanobacterium sp. MB1
BAI67102 Methanobacterium palustre *
BAI94570 Methanobacterium kanagiense *
BAI67094 Methanobacterium ferruginis *

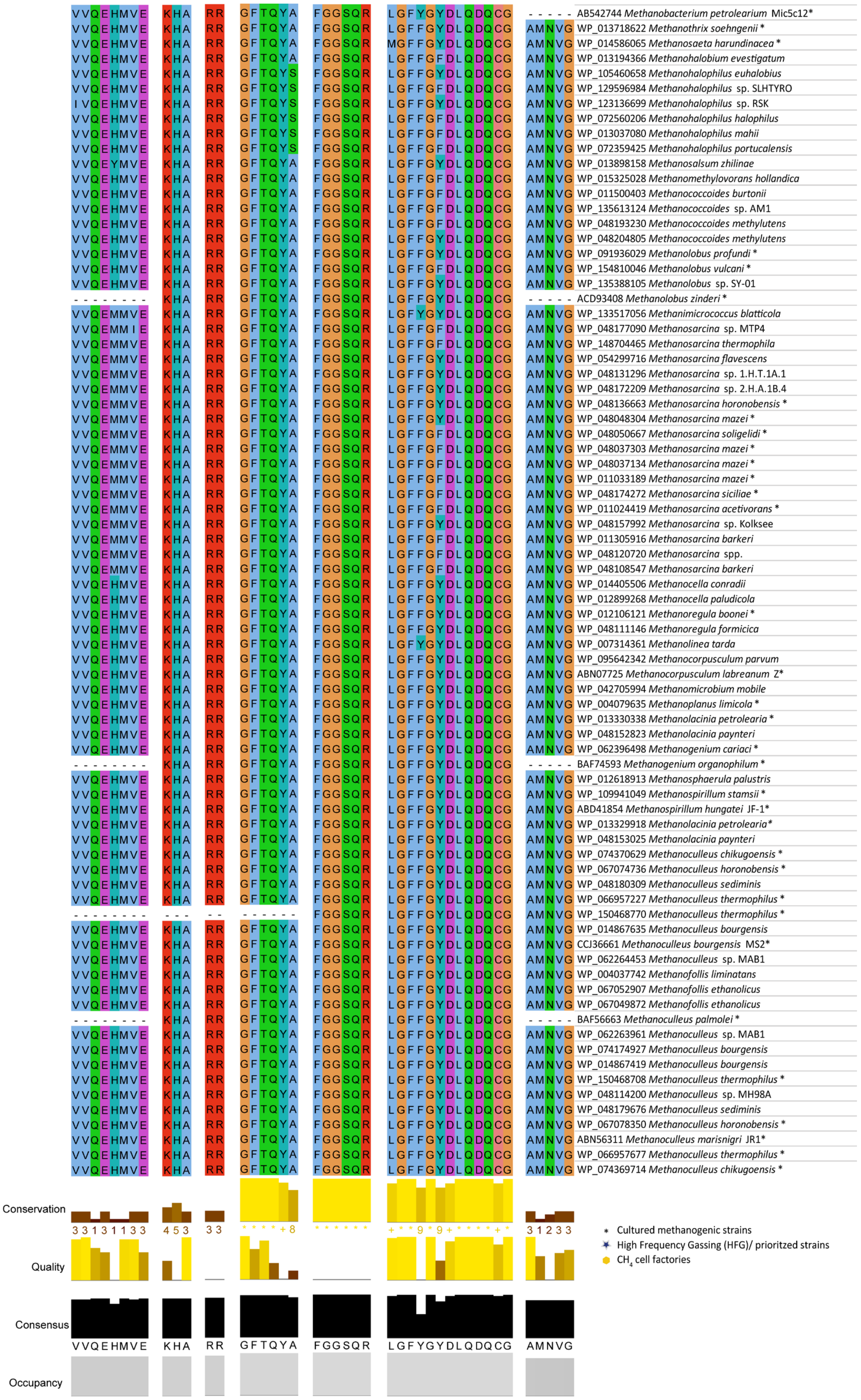

Fig. S8 Amino acid conservation within alpha subunit of the methyl-coenzyme M reductase (MCR, coenzyme-B sulfoethylthiotransferase). Blastp (Refseq, query: *M. marburgensis* Marburg, GenBank: ADL59127.1) and UniProtK data. Positions according to [3,4].

As it was shown by Borrel *et al*. 2019, *Methanonatronarchaeum thermophilum* uses asparagine instead of tyrosine on position 333 ($Tyr^{\alpha 333}$) and threonine instead of $Val^{\alpha 482}$. *Methanobrevibacter gottschalkii* shows an amino acid exchange from $Cys^{\alpha 452}$ to alanine and *Methanobacterium palustre* shows a deletion on that position indicated with X, see **Fig. S8**. It seems like, the Arg^{334} is quite conserved, but some methanogens use glycine (*Methanonatronarchaeum thermophilum*, *Methanobacterium movens*, *Methanobacterium congolense*) and serine (*Methanohalophilus* spp.) instead. *Methanosaeta harundinacea* uses methionine instead of lysine on position 441. In *M. kandleri*, an alanine replaces asparagine on position 447.

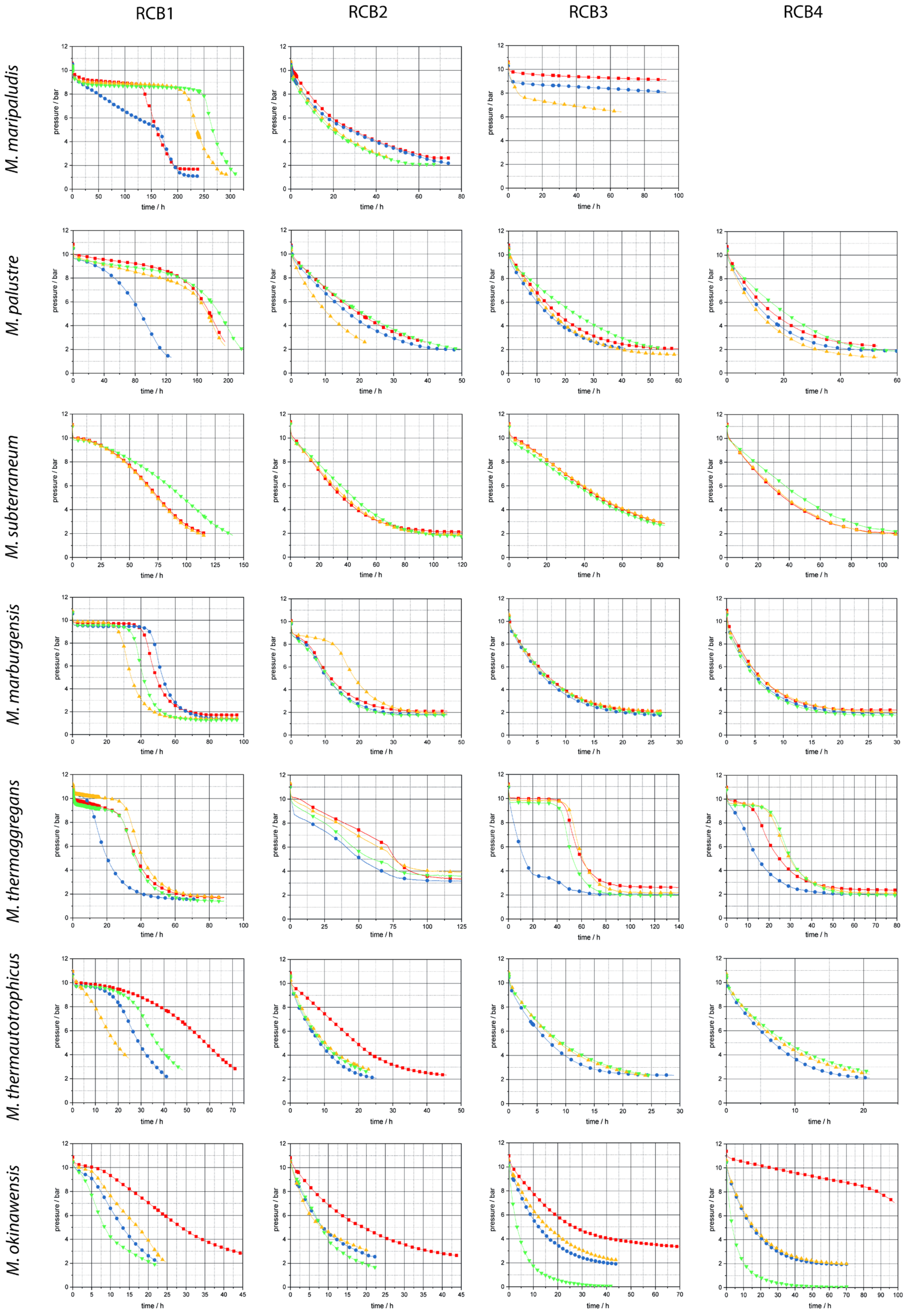

RCB1
RCB2
RCB3
RCB4
M. maripaludis
M. palustre
M. subterraneum
M. marburgensis
M. thermaggregans
M. thermautotrophicus
M. okinawensis
pressure / bar
time / h

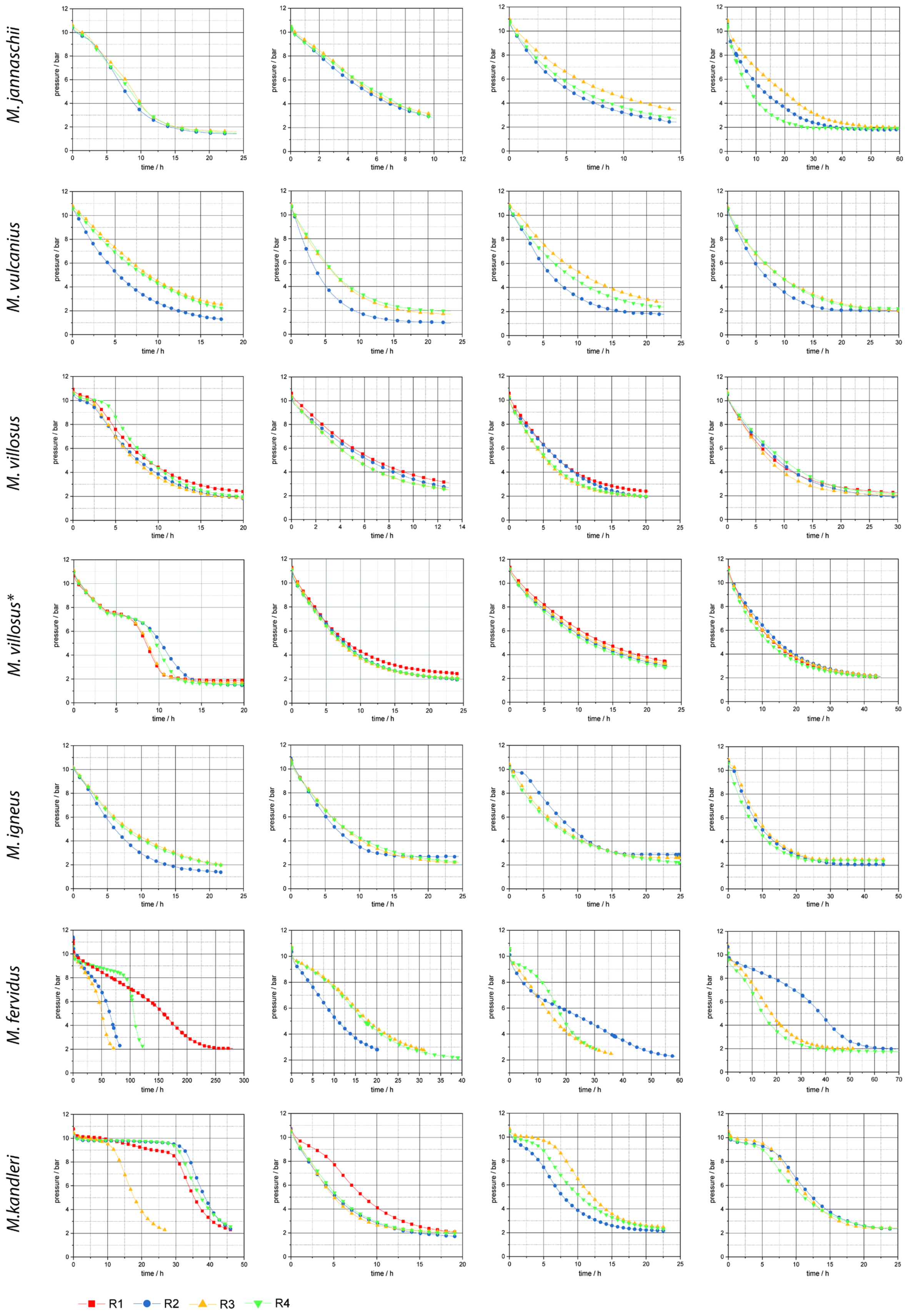

Fig. S9 Substrate uptake kinetics of twelve methanogens in RCB cultivation mode in SBRS at 10 bar.

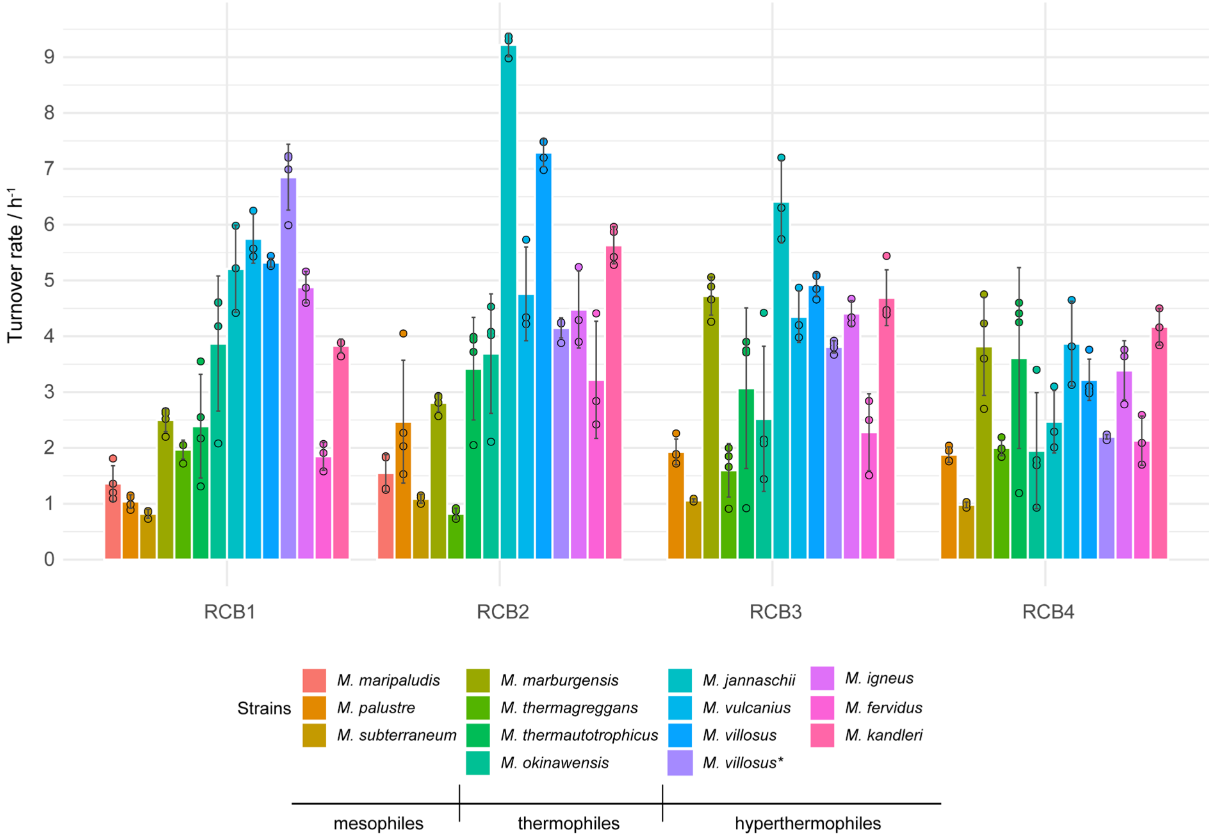

Fig. S10 Repetitive closed batch (RCB) high pressure cultivations of prioritized methanogens in the simultaneous bioreactor system (SBRS) at 10 bar H_2/CO_2 (4:1) atmosphere. All experiments were performed in quadruplicates in the SBRS system[5]. The principle of the cultivation was to repressurize each of the bioreactors to 10 bar after full headspace gas conversion. RCB1, RCB2, RCB3, and RCB4 indicate results from individual and successive closed batch headspace gas conversions. The turnover rate / h^{-1} is shown. Legend: the left block indicates mesophilic methanogens grown at 37°C (*Methanococcus maripaludis* S2, *Methanobacterium palustre* F, *Methanobacterium subterraneum* A8p); the middle block shows the thermophilic methanogens grown at 65°C (*Methanothermobacter marburgensis* Marburg, *Methanobacterium thermaggregans*, *Methanothermobacter thermautotrophicus* DeltaH, *Methanothermococcus okinawensis* IH1); the right block shows hyperthermophilic methanogens (*Methanocaldococcus jannaschii* JAL-1(80°C), *Methanocaldococcus vulcanius* M7 (80°C), *Methanocaldococcus villosus* KIN24-T80 (80°C), *Methanocaldococcus villosus* KIN24-T80*-grown on 282c 18_E medium (80°C), *Methanotorris igneus* Kol 5 (85°C), *Methanothermus fervidus* H9 (80°C), *Methanopyrus kandleri* AV19 (98°C)).

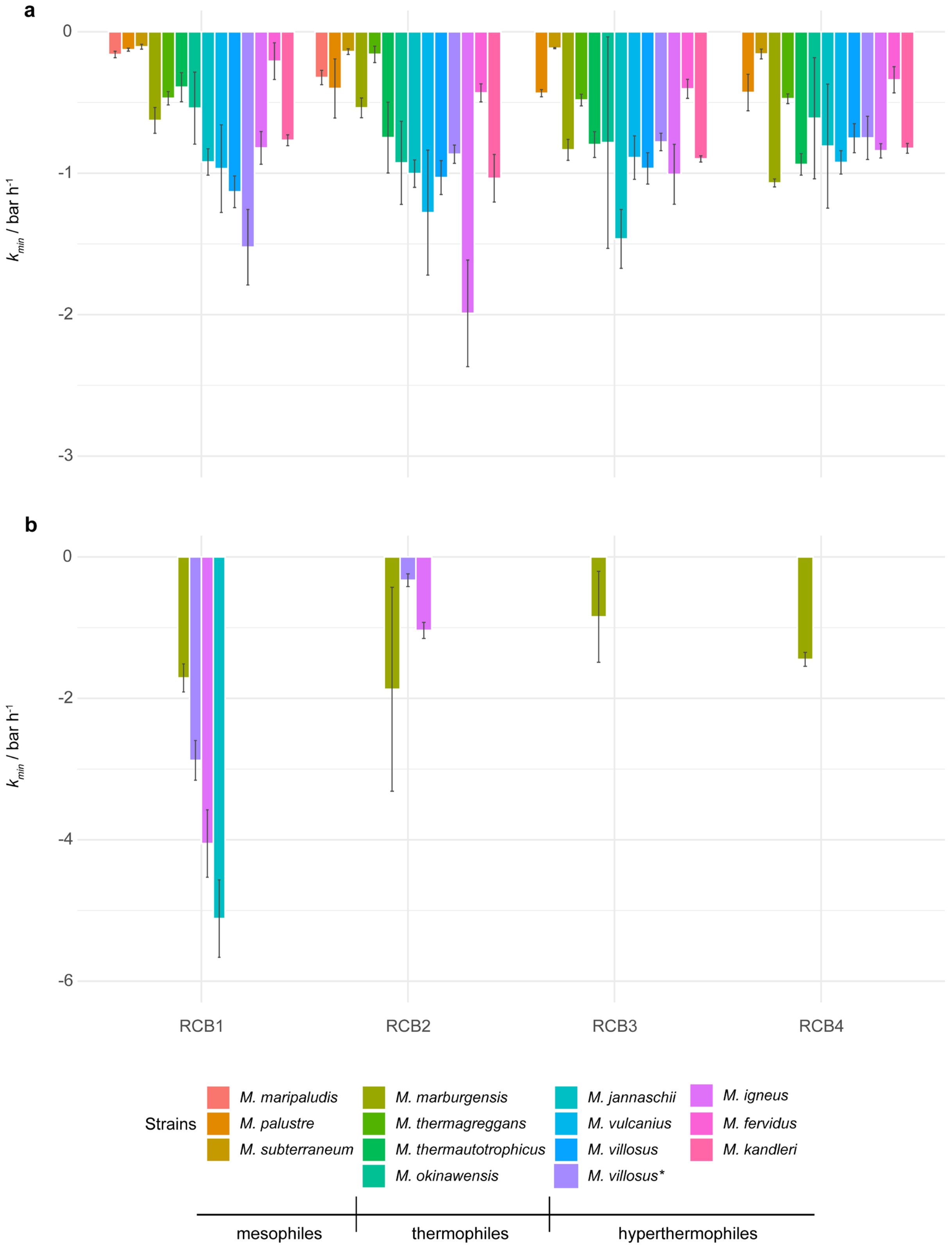

Fig. S11 Maximum negative slope k_{min} / bar h^{-1} of RCB cultivations in the SBRS at a) 10 bar and b) 50 bar.

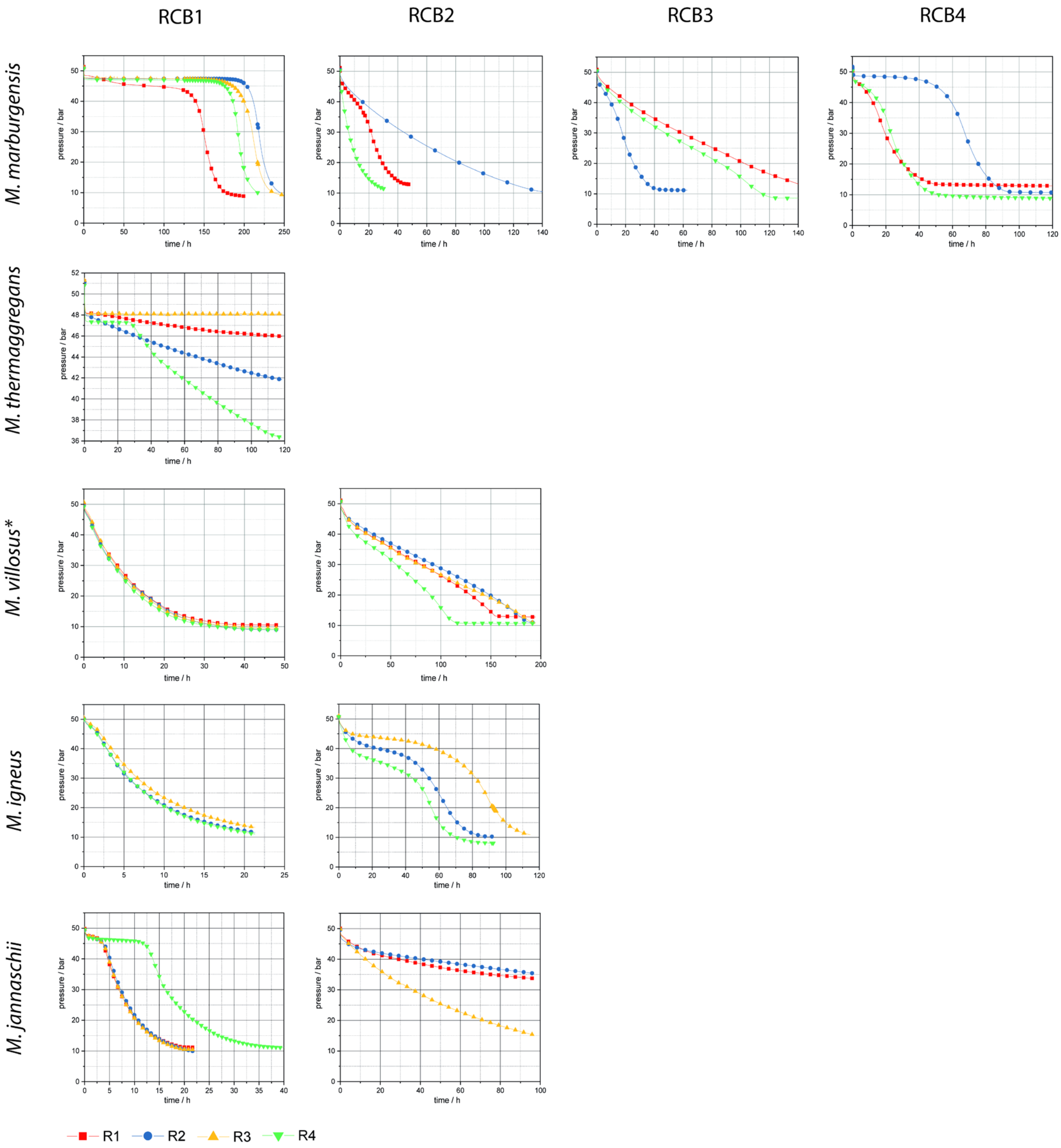

Fig. S12 Substrate uptake kinetics showing repetetive closed batch (RCB) cultivations in the SBRS at 50 bar

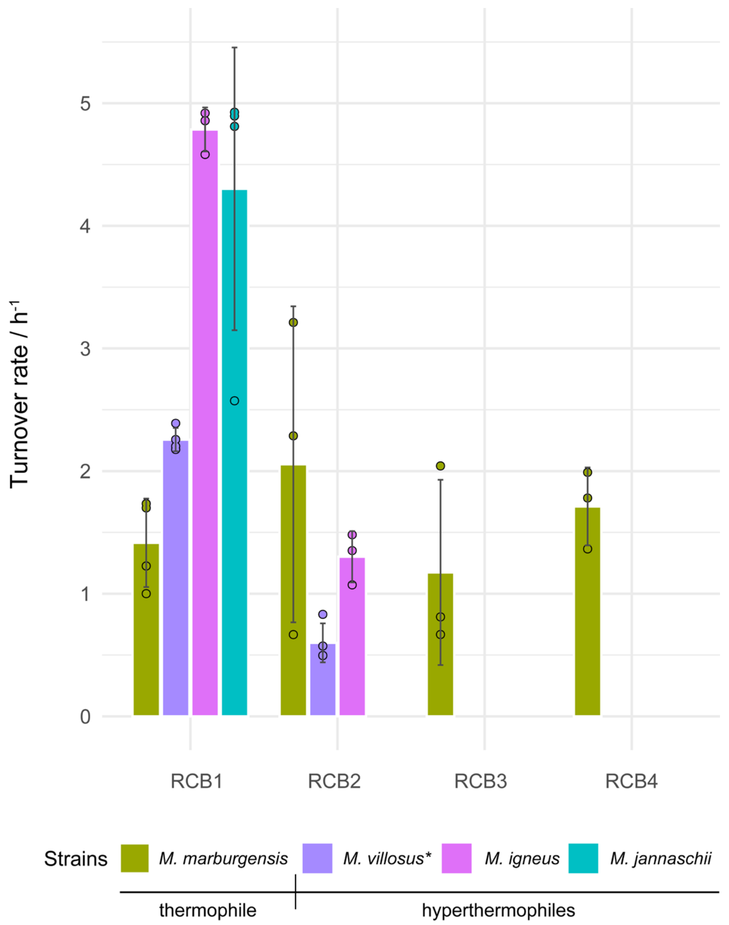

Fig. S13 Repetitive closed batch cultivations of thermophilic and hyperthermophilic methanogens in the SBRS at 50 bar. The RCB cultivations were performed in quadruplicates with 4 runs RCB1, RCB2, RCB3, and RCB4. Turnover rate / h^{-1} is shown. Legend: thermophile- *Methanothermobacter marburgensis* Marburg (65°C, MM medium); hyperthermophiles- *Methanocaldococcus jannaschii* JAL-1 (80°C, 282c 30 medium), *Methanocaldococcus villosus* KIN24-T80 (80°C, 282c18_E medium*) and *Methanotorris igneus* Kol 5 (85°C, 282c 30 medium).

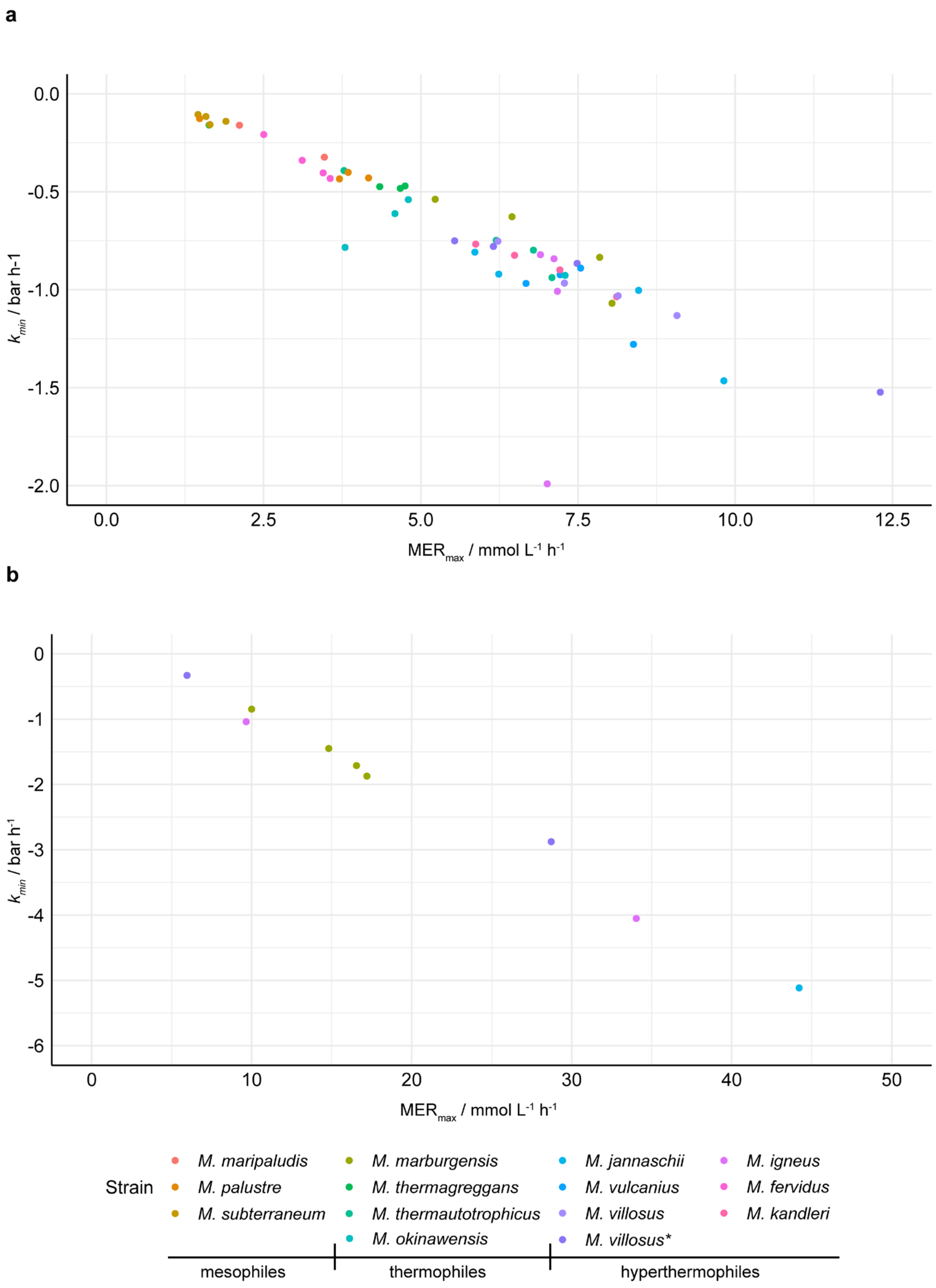

Fig. S14 Correlation plot of maximum conversion rate k_{min} / bar h^{-1} and MER_{max} / mmol L^{-1} h-1 of RCB cultivations in the SBRS at (a) 10 bar and (b) 50 bar.

Reactivation of methanogens after dormacy

It was examined how to store methanogens for fast reactivation from a state of dormancy. In case of failure due to e.g., viral infections, leading to a breakdown of the population or due to contaminations, it is of importance to have back-up cultures for fast reactivation from a state of dormancy. Therefore, studies on the storage of methanogens are inevitable to the successful functioning of an industrial bioprocess. In the microbiological lab, storing microbes in the fridge or freezer is common practice. After the pre-screening we analyzed how fast the vitality (OD and turnover) of mesophilic and (hyper-)thermophilic methanogens can be restored after they were in dormancy at 4°C or -80°C for a defined period. Our results show, that thermophilic (14 weeks at 4°C) and hyperthermophilic (>1 year at -80°C) methanogens possess a 50% higher turnoveraverage (60-88%) after dormancy compared to mesophilic methanogens (Supplementary Materials Table S1).

Table S1 Dormancy study with mesophilic, thermophilic and hyperthermophilic methanogens

Methanogens	DSMZ	Medium	Growth temp.	Dormancy Period		Turnover	$OD_{average}$	Lowest pressure	Growth after dormancy
			°C	weeks (+4°C)	years (-80°C)	%	578nm	bar	
Methanobacterium lacus **17A1**	DSM 24406	MM	30	16		2	0.04	1.916	no
Methanobacterium petrolearium **Mic5c12T**	DSM 22353	MM	35	25		15	-	1.376	yes
Methanobacterium bryantii **M.o.H.**	DSM 863	MM	37	25		1	-	1.644	no
Methanobacterium palustre **F**	DSM 3108	282c 0	37	71		56	0.28	0.632	yes
		MM		41		18	0.10	1.597	yes
		MM30		41		5	0.02	1.910	no
Methanobacterium subterraneum**A8p**	DSM 11074	MM	37	25		81	0.28	0.016	yes
		MM30		25		22	0.31	1.355	yes
Methanobacterium arcticum **M2**	DSM 19844	MM	37	25		1	-	1.683	no
Methanosarcina sicliae **T4/M**	DSM 3028	MM	37	66		1	-	1.964	no
		MM30	37	66		0	-	1.985	no
Methanothermobacter thermophilus **M**	DSM 6529	MM	60	14		85	0.34	-0.078	yes
Methanobacterium thermaggregans	DSM 3266	MM	65	14		86	0.23	-0.107	yes
Methanothermobacter marburgensis **Marburg**	DSM 2133	MM	65	14		88	0.51	-0.159	yes
Methanothermobacter thermautotrophicus **RMAS**	DSM 23052	MM	65	14		45	0.20	0.745	yes
Methanothermobacter wolfeii	DSM 2970	MM	65		7		0.25	-	yes
Methanothermococcus okinawensis **IH1**	DSM 14208	282c 18	65		1	60	0.85	0.499	yes
Methanocaldococcus jannaschii **JAL-1**	DSM 2661	282c 30	80		1	79	0.80	-0.164	yes
Methanocaldococcus villosus **KIN24-T80**	DSM 22612	282c 30	80		1	84	0.13	-0.239	yes
Methanocaldococcus vulcanius **M7**	DSM 12094	282c 30	80		1	84	0.50	-0.265	yes

Table S2 Cysteine requirements in the media of thermophilic and hyperthermophilic methaogens and the presence of cysteine biosynthesis machinery SepRS/SepCysS and cysteine metabolizing enzymes

Strains	Class	SepRS/ SepCysS	CDD	CDS	Cysteine addition	References
Methanocaldococcus jannaschii	Methanococci	+	+	-	+	11,12
Methanocaldococcus vulcanius	Methanococci	+	n.d.	n.d.	+	11
Methanotorris igneus	Methanococci	n.d.	+	-	+	11,12
Methanopyrus kandleri	Methanopyrus	+	-	+	- (vitamins)	11,12
Methanothermus fervidus	Methanobacteria	n.d.	-	+	+	12
Methanothermobacter marburgensis	Methanobacteria	n.d.	-	+	-	11,12
Methanothermobacter thermautotrophicus	Methanobacteria	+	-	+	-	11–13

n.d. not determinded

SepRS/SepCysS- cysteine production via t-RNA dependent pathway[12,14]

Cysteine desulphidase (CDD)- cysteine and water are metabolized to H_2S, NH_4^+, H^+ and pyruvate[15]

Cysteine desulphurase (CSD)- cysteine is processed to L-alanine and is bound to the enzyme-S-sulfanylcysteine[16]

Table S3 Core lipids of prioritized methanogens

Strains	Core lipids				Reference
	Archaeol	Tetraether lipids	Hydroxyarchaeol	Macrocyclic archaeol	
***Methanococuss maripaludis* S2**	+	nd.	+	-	[17–19]
***Methanobacterium palustre* F**	+	+	-	-	[17–19]
***Methanobacterium subterraneum* A8p**	+	+	-	-	[17–19]
***Methanothermobacter marburgensis* Marburg**	+	GDGT-0 [a]	-	-	[17–21]
***Methanothermobacter thermophilus* M**	+	+	-	-	[17–19]
**Methanobacterium thermaggregans**	+	+	-	-	[17–19]
***Methanothermobacter thermautotorphicus* DeltaH**	+	GDGT<br>75-83 mol%	-	-	[17–21]
***Methanothermococcus okinawensis* IH1**	59% (mostly monocyclic)	3.7% GDGT-0<br>1% GMGT-0<br>0.4% GMGT-0´,<br>0.2% GTGT-0	-	35% (mostly monocyclic)	[19,22,23]
***Methanocaldococcus jannaschii* JAL-1 [b, g]**	18 %[c]	36%	-	46%	[17–19,24]
	7 %[d]	52%		41%	
	0–2 %[e]	65%		35%	
	0–2 %[f]	64%		36%	
***Methanocaldococcus vulcanius* M7**	+	+	-	+	[17–19]
***Methanocaldococcus villosus* KIN24-T80**	39% (mostly dicyclic)	4.3% GDGT-0<br>0.1% GMGT-0<br>1% GMGT-0´<br>0.2% GTGT-0	-	55% (mostly dicyclic)	[19,23]
***Methanotorris igneus* Kol5**	+	GDGT-0	-	+	[17,18,25]
***Methanothermus fervidus* H9**	+	GDGT-0<br>GTGT/ H-GDGT	-	-	[17,21]
***Methanopyrus kandleri* AV19**	+ [h]	GDGT-0,<br>GDGT-1,<br>GDGT-2,<br>GDGT-3,<br>GDGT-4,<br>GTGT/<br>H-GDGT	+	-	[17–19,21]

a growth with detergent, increased caldarchaeols[26], b archaeols decline and caldarchaeols, macrocyclic archaeol polar lipids, increase as temperature and pressure rise[24]
c 1 atm 1 bar and 75°C; d 1 atm 1 bar and 86°C; e 250 atm 253 bar and 86°C; f 500 atm 506 bar and 86°C, g mostly GDGT-0, h unsaturated archaeol-allyl ether type core lipids- geranylgeranyl group-containing archaeol

Supplementary references

1. Bryant, M. P., Wolin, E. A., Wolin, M. J. & Wolfe, R. S. *Methanobacillus omelianskii*, a symbiotic association of two species of bacteria. *Archiv für Mikrobiologie* **59,** 20–31 (1967).
2. Balch, W. E., Fox, G. E., Magrum, L. J., Woese, C. R. & Wolfe, R. S. Methanogens: reevaluation of a unique biological group. *Microbiological Reviews* **43,** 260–296 (1979).
3. Ermler, U., Grabarse, W., Shima, S., Goubeaud, M. & Thauer, R. K. Crystal structure of methyl-coenzyme M reductase: the key enzyme of biological methane formation. *Science* **278,** 1457–1462 (1997).
4. Borrel, G., Adam, P. S., McKay, L. J., Chen, L.-X., Sierra-García, I. N., Sieber, C. M., Letourneur, Q., Ghozlane, A., Andersen, G. L. & Li, W.-J. Wide diversity of methane and short-chain alkane metabolisms in uncultured archaea. *Nature microbiology* **4,** 603–613 (2019).
5. Pappenreiter, P. A., Zwirtmayr, S., Mauerhofer, L.-M., Rittmann, S. K.-M. R. & Paulik, C. Development of a simultaneous bioreactor system for characterization of gas production kinetics of methanogenic archaea at high pressure. *Engineering in Life Sciences* **19,** 537–544 (2019).
6. UniProt: a worldwide hub of protein knowledge. *Nucleic Acids Res* **47,** D506–D515 (2019).
7. El-Gebali, S., Mistry, J., Bateman, A., Eddy, S. R., Luciani, A., Potter, S. C., Qureshi, M., Richardson, L. J., Salazar, G. A., Smart, A., Sonnhammer, E. L. L., Hirsh, L., Paladin, L., Piovesan, D., Tosatto, S. C. E. & Finn, R. D. The Pfam protein families database in 2019. *Nucleic Acids Res* **47,** D427–D432 (2019).
8. Bellack, A. Cell architecture and flagella of hyperthermophilic Archaea. (2011).
9. Bellack, A., Huber, H., Rachel, R., Wanner, G. & Wirth, R. *Methanocaldococcus villosus* sp. nov., a heavily flagellated archaeon that adheres to surfaces and forms cell–cell contacts. *International journal of systematic and evolutionary microbiology* **61,** 1239–1245 (2011).
10. König, H., Rachel, R. & Claus, H. in *Archaea* 315–340 (American Society of Microbiology, 2007).
11. Hidese, R., Inoue, T., Imanaka, T. & Fujiwara, S. Cysteine desulphurase plays an important role in environmental adaptation of the hyperthermophilic archaeon *Thermococcus kodakarensis*. *Molecular Microbiology* **93,** 331–345 (2014).
12. Liu, Y., Sieprawska-Lupa, M., Whitman, W. B. & White, R. H. Cysteine Is Not the Sulfur Source for Iron-Sulfur Cluster and Methionine Biosynthesis in the Methanogenic Archaeon *Methanococcus maripaludis*. *Journal of Biological Chemistry* **285,** 31923–31929 (2010).
13. Kärcher, U., Schröder, H., Haslinger, E., Allmaier, G., Schreiner, R., Wieland, F., Haselbeck, A. & König, H. Primary structure of the heterosaccharide of the surface glycoprotein of *Methanothermus fervidus*. *Journal of Biological Chemistry* **268,** 26821–26826 (1993).
14. Klipcan, L., Frenkel-Morgenstern, M. & Safro, M. G. Presence of tRNA-dependent pathways correlates with high cysteine content in methanogenic Archaea. *Trends in Genetics* **24,** 59–63 (2008).
15. Tchong, S.-I., Xu, H. & White, R. H. l-Cysteine Desulfidase: An [4Fe-4S] Enzyme Isolated from *Methanocaldococcus jannaschii* That Catalyzes the Breakdown of l-Cysteine into Pyruvate, Ammonia, and Sulfide. *Biochemistry* **44,** 1659–1670 (2005).
16. Zheng, L., White, R. H., Cash, V. L., Jack, R. F. & Dean, D. R. Cysteine desulfurase activity indicates a role for NIFS in metallocluster biosynthesis. *Proc Natl Acad Sci USA* **90,** 2754 (1993).
17. Koga, Y., Nishihara, M., Morii, H. & Akagawa-Matsushita, M. Ether polar lipids of methanogenic bacteria: structures, comparative aspects, and biosyntheses. *Microbiology and Molecular Biology Reviews* **57,** 164–182 (1993).
18. Koga, Y., Morii, H., Akagawa-Matsushita, M. & Ohga, M. Correlation of Polar Lipid Composition with 16S rRNA Phylogeny in Methanogens. Further Analysis of Lipid Component Parts. *Bioscience, Biotechnology, and Biochemistry* **62,** 230–236 (1998).
19. Koga, Y. & Morii, H. Recent Advances in Structural Research on Ether Lipids from Archaea Including Comparative and Physiological Aspects. *Bioscience, Biotechnology, and Biochemistry* **69,** 2019–2034 (2005).
20. Knappy, C. S. Mass spectrometric studies of ether lipids in Archaea and sediments. (2010).
21. Schouten, S., Hopmans, E. C. & Sinninghe Damsté, J. S. The organic geochemistry of glycerol dialkyl glycerol tetraether lipids: A review. *Organic Geochemistry* **54,** 19–61 (2013).
22. Taubner, R.-S., Baumann, L. M., Bauersachs, T., Clifford, E. L., Mähnert, B., Reischl, B., Seifert, R., Peckmann, J., Rittmann, S. K.-M. & Birgel, D. Membrane Lipid Composition and Amino Acid Excretion Patterns of *Methanothermococcus okinawensis* Grown in the Presence of Inhibitors Detected in the Enceladian Plume. *Life* **9,** 85 (2019).
23. Baumann, L. M. F., Taubner, R.-S., Bauersachs, T., Steiner, M., Schleper, C., Peckmann, J., Rittmann, S. K.-M. R. & Birgel, D. Intact polar lipid and core lipid inventory of the hydrothermal vent methanogens *Methanocaldococcus villosus* and *Methanothermococcus okinawensis*. *Organic Geochemistry* **126,** 33–42 (2018).
24. Kaneshiro, S. M. & Clark, D. S. Pressure effects on the composition and thermal behavior of lipids from the deep-sea thermophile *Methanococcus jannaschii*. *J. Bacteriol.* **177,** 3668 (1995).

25. Trincone, A., Nicolaus, B., Palmieri, G., De Rosa, M., Huber, R., Huber, G., Stetter, K. O. & Gambacorta, A. Distribution of complex and core lipids within new hyperthermophilic members of the Archaea domain. *Systematic and applied microbiology* **15,** 11–17 (1992).
26. Gräther, O. W., Arigoni, D. & Jaun, B. Zur Struktur und Biosynthese der Tetraetherlipide der Archaea. (1994). doi:10.3929/ethz-a-001377425
