# Supplementary material for: Hyperthermophilic methanogenic archaea act as high-pressure CH4 cell factories
Source: Commun Biol. 2021 Mar 5;4:289. doi: 10.1038/s42003-021-01828-5 (PMC7935968; doi:10.1038/s42003-021-01828-5)
Supplement: Supplementary file 3 — Description of Additional Supplementary Files [file 42003_2021_1828_MOESM3_ESM.pdf]

Description of Additional Supplementary Files

File Name: Supplementary Data 1

Description: Supplementary data and information to the manuscript entitled *Hyperthermophilic methanogenic archaea act as high-pressure CH4 cell factories*

File Name: Supplementary Data 2

Description: Supplementary data about the cell envelope characteristics of prioritized methanogens with respect to S-layer occurrence to the manuscript entitled *Hyperthermophilic methanogenic archaea act as high-pressure CH4 cell factories*
